# Excitatory neuron-prone prion propagation and excitatory neuronal loss in prion-infected mice

**DOI:** 10.3389/fnmol.2024.1498142

**Published:** 2024-12-12

**Authors:** Temuulen Erdenebat, Yusuke Komatsu, Nozomi Uwamori, Misaki Tanaka, Takashi Hoshika, Takeshi Yamasaki, Ayano Shimakura, Akio Suzuki, Toyotaka Sato, Motohiro Horiuchi

**Affiliations:** ^1^Laboratory of Veterinary Hygiene, Faculty of Veterinary Medicine, Graduate School of Infectious Diseases, Hokkaido University, Sapporo, Japan; ^2^One Health Research Center, Hokkaido University, Sapporo, Japan; ^3^Global Station for Zoonosis Control, Global Institute for Collaborative Research and Education, Hokkaido University, Sapporo, Japan

**Keywords:** prion, PrP^Sc^, excitatory neuron, inhibitory neuron, glutamatergic neuron, GABAergic neuron

## Abstract

The accumulation of a disease-specific isoform of prion protein (PrP^Sc^) and histopathological lesions, such as neuronal loss, are unevenly distributed in the brains of humans and animals affected with prion diseases. This distribution varies depending on the diseases and/or the combinations of prion strain and experimental animal. The brain region-dependent distribution of PrP^Sc^ and neuropathological lesions suggests a neuronal cell-type-dependent prion propagation and vulnerability to prion infection. However, the underlying mechanism is largely unknown. In this study, we provided evidence that the prion 22L strain propagates more efficiently in excitatory neurons than inhibitory neurons and that excitatory neurons in the thalamus are vulnerable to prion infection. PrP^Sc^ accumulation was less intense in the striatum, where GABAergic inhibitory neurons predominate, compared to the cerebral cortex and thalamus, where glutamatergic excitatory neurons are predominant, in mice intracerebrally or intraperitoneally inoculated with the 22L strain. PrP^Sc^ stains were observed along the needle track after stereotaxic injection into the striatum, whereas they were also observed away from the needle track in the thalamus. Consistent with inefficient prion propagation in the striatum, the 22L prion propagated more efficiently in glutamatergic neurons than GABAergic neurons in primary neuronal cultures. RNAscope *in situ* hybridization revealed a decrease in *Vglut1*- and *Vglut2*-expressing neurons in the ventral posterolateral nuclei of the thalamus in 22L strain-infected mice, whereas no decrease in *Vgat*-expressing neurons was observed in the adjacent reticular nucleus, mainly composed of *Vgat*-expressing interneurons. The excitatory neuron-prone prion propagation and excitatory neuronal loss in 22L strain-infected mice shed light on the neuropathological mechanism of prion diseases.

## Introduction

Prion diseases are fatal and intractable neurodegenerative disorders affecting both animals and humans, including bovine spongiform encephalopathy, scrapie in sheep and goats, chronic wasting disease in cervids, and Creutzfeldt-Jakob disease (CJD) in humans. These diseases have long incubation periods but become subacutely progressive and inevitably fatal after clinical onset. Neuropathological hallmarks include astrogliosis, microglial activation, neuronal and neuropil vacuolation, neuronal loss, and deposition of a disease-specific prion protein (PrP^Sc^) in the central nervous systems (CNS) (Collinge, 2001; Prusiner, 1998). PrP^Sc^ is generated from a cellular isoform of prion protein (PrP^C^), encoded by the host *Prnp* gene, through post-translational conformational transition. PrP^Sc^, rich in *β*-sheets and prone to aggregation, is considered the sole proteinaceous component of prions, the infectious agents of prion diseases. Prion infectivity is believed to be associated with PrP^Sc^ oligomers/aggregates (Silveira et al., 2005; Wang et al., 2010).

PrP^Sc^ deposition and neurohistopathological lesions, such as neuronal and neuropil vacuolation, are observed in various brain regions of prion-infected mice. However, the distribution varies depending on the prion and mouse strain combinations (Bruce et al., 1994; Bruce, 1993; DeArmond et al., 1993). For example, intense PrP^Sc^ accumulation in the thalamus and cerebral cortex has been reported in multiple prion strains, including the 22L strain (Carroll et al., 2016; Reis et al., 2015). Some mouse-adapted strains from Gerstmann-Sträussler-Scheinker syndrome and Fatal Familial Insomnia show intense PrP^Sc^ accumulation in the thalamus but mild accumulation in the cerebral cortex (Tateishi et al., 1995; Telling et al., 1996). The prion strain-dependent PrP^Sc^ distribution in the CNS suggests neuronal cell-type tropism or differences in prion propagation efficiency among neuronal cell types, though the underlying mechanism remains largely unknown. Neuronal loss is not solely due to prion propagation in neurons; innate immune responses, such as microglial activation and astrogliosis, are deeply involved in prion disease neuropathology (Carroll et al., 2018; Gómez-Nicola et al., 2013; Makarava et al., 2020; Manuelidis et al., 1987; Mukherjee et al., 2010). Prion propagation in neurons is believed to trigger innate immune responses, similar to the early microglial response to Amyloid *β* deposition (Wang et al., 2015), and is essential for neuronal degeneration (Chesebro et al., 2005; Mallucci et al., 2003). Therefore, understanding the mechanism behind brain region-dependent prion propagation is crucial for understanding the neuropathophysiology of prion diseases.

In mammals, two neurotransmitters, glutamate and gamma-aminobutyric acid (GABA), predominantly regulate neuronal activity in the brain. Glutamate is synthesized in excitatory glutamatergic neurons and astrocytes, while GABA is synthesized in GABAergic inhibitory neurons (Fremeau et al., 2004b; Tepper et al., 2018). The distribution of glutamatergic and GABAergic neurons varies across brain regions. In the cerebral cortex, approximately 10–20% of neurons are inhibitory (Rudy et al., 2011; Sahara et al., 2012; Tremblay et al., 2016), whereas GABAergic inhibitory neurons account for over 90% of neurons in the striatum (Assous et al., 2018; Tepper et al., 2004). The major neurons in the thalamus are glutamatergic, except for most lateral nuclei (Fremeau et al., 2004a; Fremeau et al., 2004b; Pinault, 2004). Specific marker genes for these neuronal types have been identified and are used to distinguish neuronal cell types. Glutamatergic neurons are characterized by the expression of vesicular glutamate transporters, Vglut1, Vglut2, and Vglut3 (Vgluts), which localize to synaptic vesicles and accumulate L-glutamate (Fremeau et al., 2001; Gras et al., 2002). Similarly, GABAergic neurons are identified by the presence of the vesicular GABA transporter (Vgat) (Chaudhry et al., 1998).

Excitatory and inhibitory neurons have different developmental lineages and numerous molecular and morphological differences (Zhang et al., 2021), which may affect their efficiency in prion propagation and vulnerability to prion infection. The selective loss of parvalbumin-positive GABAergic inhibitory neurons in the brains of CJD patients and prion-infected mice suggests that GABAergic neurons are vulnerable to prion infection (Belichenko et al., 1999; Guentchev et al., 1997; Guentchev et al., 1999). Recent single-cell transcriptome analysis indicated that inhibitory neurons in the hippocampus of RML prion-infected mice are more vulnerable to prion infection (Slota et al., 2022). However, non-GABAergic neurons degenerated earlier than GABAergic neurons and the degeneration of GABA-negative synapses was observed before the degeneration of GABAergic neurons in prion-infected hamsters (Bouzamondo et al., 2000; Bouzamondo-Bernstein et al., 2004). Additionally, selective loss of Vglut1-positive synapses in the cerebellum and pontine nuclei was observed in BSE-infected guinea pigs (Sakaguchi et al., 2020). The single-cell transcriptome analysis also found differentially expressed transcripts from the excitatory neuron populations in the RML prion-infected mice, suggesting synaptic disfunction of excitatory neurons (Slota et al., 2022).

Our preliminary results showed weaker PrP^Sc^ accumulation in the striatum of prion-infected mice compared to the cerebral cortex and thalamus (Supplementary Figure 1). This brain region-dependent PrP^Sc^ accumulation may be related to the striatum composition, which is largely GABAergic neurons (Tepper et al., 2004). We hypothesized that a deeper analysis of this phenomenon would shed light on the neuropathological mechanisms related to neuronal cell-type-dependent prion propagation and cell death in prion diseases. Therefore, we analyzed brain region-dependent PrP^Sc^ accumulation in prion-infected mice using *in vivo* and primary neuronal cultures. We found that 22L prions propagate more efficiently in glutamatergic excitatory neurons than in GABAergic inhibitory neurons, consistent with the lower PrP^Sc^ accumulation in the striatum. This neuronal cell-type-prone prion propagation will enhance our understanding of prion disease neuropathology.

## Materials and methods

### Ethics statement and animals

All mice were housed at the Faculty of Veterinary Medicine, Hokkaido University, in an AAALAC-accredited facility, following guidelines from the Institutional Animal Care and Use Committee (approval No. 23-0041). Jcl:ICR mice were purchased from CLEA Japan (Tokyo, Japan) and acclimated for a week. They had unrestricted access to food and water under a 12-h light/dark cycle.

### Prion strains

Mouse-adapted prion 22L, Chandler, and Obihiro strains were used. Brains from terminal-stage diseased and age-matched mock-infected mice were used to prepare 10% (w/v) brain homogenates in phosphate-buffered saline (PBS) as stock solutions.

### Intracerebral, intraperitoneal, and stereotaxic inoculation

Four-week-old female Jcl:ICR mice were anesthetized with sevoflurane (Maruishi Pharmaceutical, Osaka, Japan) and inoculated either intracerebrally (i.c.) with 20 μL of 2.5% brain homogenates or intraperitoneally (i.p.) with 200 μL of 1.0% brain homogenates from prion-infected or age-matched mock-infected mice. In intracerebral inoculation, brain homogenates (20 μL) were inoculated into the left hemisphere with two-stage needle of 27-gauge under anesthesia with sevoflurane.

For stereotaxic inoculation, eight-week-old female ICR mice were anesthetized with an intramuscular injection of xylazine (10 mg/kg) and ketamine (100 mg/kg), then placed on a stereotaxic apparatus (Narishige, Tokyo, Japan). A 1 cm midline incision was made on the dorsal skull surface to expose the skull and position the drill over the Bregma point. Coordinates for targeting the right thalamus were anteroposterior (AP) −1.7 mm, medial-lateral (ML), −1.5 mm from Bregma, and 4 mm depth; for the left striatum, AP 1 mm, ML 1.6 mm, and 4 mm depth. Coronal sections for the thalamus and striatum were at Plates 45–46 (Bregma between −1.67 and −1.79 mm) and 22–23 (Bregma between 0.97 and 1.09 mm), respectively (Paxinos and Franklin, 2013). These coordinates avoided ventricles. Brain homogenates (0.5 μL, 0.1% in PBS) from prion 22L strain-infected or age-matched mock-infected mice were stereotaxically inoculated into the thalamus or striatum. Injections were performed with Hamilton syringes (#80301, Hamilton Company, Reno, NV, USA) and 31-gauge steel bevel needles (#7750–22 Hamilton Company) at 0.062 μL/min using a microinjection pump (#78–8130, KD Scientific, Holliston, MA, USA). The needle remained in place for 2 min post-injection to prevent backflow. The skin incision was closed with a synthetic absorbable suture (#D6284, Ethicon, Raritan, NJ, USA). Needle patency was confirmed before and after injections.

### Tissue processing and histology

Mice were euthanized under anesthesia with sevoflurane and their brains were taken and rinsed with PBS, embedded in Tissue-Tek O.C.T. compound (#4583, Sakura Finetek, Torrance, CA, USA) for frozen sections, and stored at −80°C. Ten μm sagittal plane frozen sections were cut using a cryostat (#Leica CM1950, Leica, Heidelberger Straße, Germany) and mounted on slide glasses (#APS-02, Matsunami, Osaka, Japan). For formalin-fixed paraffin-embedded (FFPE) sections, brains were fixed in 10% neutral buffered formalin (#062–01661, Wako, Osaka, Japan) for 5 days. Whole brains were then cut at the coronal plane to include the entire striatum or thalamus, dehydrated, and embedded in paraffin. FFPE sections were cut into 4 μm slices using a microtome and mounted on slide glasses (#APS-02, Matsunami). Sections were stained with hematoxylin (#30022, Carazzi’s Hematoxylin, Muto Pure Chemicals, Tokyo, Japan) and eosin (#32002, 1% Eosin Y Solution, Muto Pure Chemicals) or subjected to immunohistochemistry (IHC) for PrP^Sc^ detection.

### Immunohistochemistry (IHC)

The sections were deparaffinized, rehydrated, and autoclaved at 135°C for 30 min for PrP^Sc^ detection (Song et al., 2008). They were then treated with 3% H_2_O_2_ in methanol for 5 min, blocked with 5% fetal bovine serum (FBS) in PBS for 30 min at room temperature (r.t.) and incubated overnight at 4°C with anti-PrP monoclonal antibody (mAb) 132 (1 μg/mL). After washing with PBS, the sections were incubated for 1 h at r.t. with anti-mouse IgG F (ab)_2_ -Peroxidase antibody (1:2,000, #A3682, Sigma-Aldrich, St. Louis, MS, USA). The sections were washed with PBS and developed using the DAB Substrate Kit, Peroxidase (HRP), with Nickel (#SK-4100, Vector Laboratories, Newark, CA, USA), followed by counterstaining with hematoxylin. Finally, the sections were mounted with Mount-Quick (#Mount-Quick, Daido Sangyo, Tokyo, Japan). Observations and image acquisition were performed using the Keyence BZ-X810 Fluorescence Microscope and BZ-X800 Viewer software (Keyence, Itasca, IL, USA).

### Immunofluorescence assay (IFA) for mouse brain

The frozen brain sections were fixed with 4% paraformaldehyde (PFA) (#163–20,145, Wako) for 10 min at r.t. After washing with PBS containing 0.01% Tween 20, the sections were permeabilized with 0.1 M glycine and 0.1% Triton X-100 for 20 min at r.t., followed by incubation with 2.5 M guanidine hydrochloride for 15 min at r.t. The sections were then blocked with 5% FBS in PBS for 1 h at r.t. and incubated overnight at 4°C with Alexa Fluor 488-conjugated anti-PrP mAb 132 and anti-NeuN rabbit monoclonal antibody (1:2,000, #ab177487, Abcam, Cambridge, UK) in 1% FBS in PBS. For the secondary antibody reaction, the sections were incubated with goat anti-rabbit IgG F(ab’)_2_ Alexa Fluor 555 antibody (1:1,000, #A-21430, Thermo Fisher Scientific, Waltham, MA, USA) and counterstained with 4′,6-diamidino-2-phenylindole (DAPI) (#D3571, Thermo Fisher Scientific, 5 μg/mL in 1% FBS in PBS) for nuclei at r.t. for 1 h. Finally, the sections were mounted with Prolong Gold antifade reagent (#P36934, Thermo Fisher Scientific). Tile scan fluorescent images were acquired using a laser scanning confocal microscope LSM700 (Zeiss, Oberkochen, Germany) with a 20× objective lens.

### Primary neuronal cell cultures

In this study, thalamic (ThN), striatal (StN), and cerebral cortex neuronal cultures (CxN) were prepared from ICR mouse embryos at embryonic day 14. Pregnant mice were euthanized with sevoflurane, and the uterus containing the fetuses was removed. The brains of the fetuses were dissected in ice-cold PBS under a stereomicroscope. The medulla oblongata, hippocampi, and meninges were carefully removed, and the thalami, striata, and cerebral cortices were separately collected. Tissue dissociation, cell seeding, and inhibition of astrocyte proliferation were performed as previously described (Tanaka et al., 2016). At 7 days *in vitro* (div), primary neuronal cultures were exposed to microsomal fractions from prion 22 L-infected mouse brains, containing 5 ng rPrP equivalent proteinase K-resistant PrP (PrP-res) per 1.0 × 10^5^ cells, by replacing half the medium in each well. At 4 days post-inoculation (dpi), the medium was completely replaced with fresh neuronal medium.

### SDS-PAGE and immunoblotting

Neurons cultured on 6-well plastic plates were lysed with 300 μL/well of lysis buffer (0.5% Triton X-100, 0.5% sodium deoxycholate, 150 mM NaCl, 5 mM EDTA, and 10 mM Tris–HCl [pH 7.5]). The cells underwent one freeze–thaw cycle followed by pipetting. After centrifugation, protein concentrations of the lysates were measured using a DC protein assay kit (#5000116, Bio-Rad-Life Science, Hercules, CA, USA) and adjusted to 0.5–0.8 mg/mL. SDS-PAGE and immunoblotting for PrP-res detection were performed as previously described (Tanaka et al., 2016; Tanaka et al., 2020).

### IFA for primary neurons

PrP^Sc^-specific immunofluorescence staining of prion-infected primary neuronal cultures using anti-PrP mAb 8D5 was performed as previously described (Tanaka et al., 2016; Tanaka et al., 2020). For staining Vglut1, Vglut2, and Vgat, cells were fixed with 4% PFA in PBS at r.t. for 10 min and permeabilized with PBS containing 0.1 M glycine and 0.1% Triton X-100 at r.t. for 10 min. After blocking with 5% FBS in PBS, the cells were incubated with primary antibodies: anti-Vgat rabbit polyclonal antibody (1:4,000, #131002, Synaptic System, Göttingen, Germany), anti-Vglut1 guinea pig polyclonal antibody (1:4,000, #135304, Synaptic System), and anti-Vglut2 guinea pig polyclonal antibody (1:2,000, #135404, Synaptic System). Anti-MAP2 chicken polyclonal antibody (1:2,000, #ab5392, Abcam) was used for staining neurons. Secondary antibodies used were goat anti-chicken IgY conjugated with Alexa Fluor 647 (1:4,000, #ab150173, Abcam) for detecting MAP2, goat anti-rabbit IgG Alexa Fluor 555 (1:4,000, #A21245, Thermo Fisher Scientific) for Vgat, and goat anti-guinea pig IgG Alexa Fluor 488 (1,4,000, #A11073, Thermo Fisher Scientific) for Vglut1 and Vglut2. Nuclei were counterstained with DAPI.

The STAIN Perfect Immunostaining Kit A (#SP-A-1000, ImmuSmol, Bordeaux, France) was used to stain L-glutamate and GABA in glutamatergic and GABAergic neurons in primary neuronal cultures. Cells were fixed, permeabilized, stabilized, and saturated with reagents from the kit according to the manufacturer’s instructions. They were then incubated overnight at 4°C with primary antibodies: anti-L-glutamate antibody (1:1,000, #IS1001, ImmuSmol), anti-GABA antibody (1:1,000, #IS1036, ImmuSmol), and mAb 8D5 (1 μg/mL) in the antibody diluent. After three washes with Wash 2 solution, cells were incubated for 60 min at r.t. with secondary antibodies: goat anti-rabbit IgG conjugated with Alexa Fluor 555 antibodies (1:4,000, #A21245, Thermo Fisher Scientific) for anti-L-glutamate, goat anti-chicken IgY conjugated with Alexa Fluor 647 antibodies (1:4,000, #ab150173, Abcam) for anti-GABA, and F(ab’)_2_-goat anti-mouse IgG conjugated with Alexa Fluor 488 antibodies (1:4,000, #A-11017, Invitrogen) for PrP^Sc^ in 1% FBS-PBS. Nuclei were counterstained with DAPI.

Stained cells were observed using a Zeiss LSM800 laser scanning microscope (Zeiss) with a 20x objective lens. For higher resolution, Airyscan using highly sensitive GaAsP detectors on the Zeiss LSM800 with a 63x objective lens. Acquired images were analyzed with ZEN 2.6 software (blue edition).

### RNAscope *in situ* hybridization

The RNAscope^®^ 2.5 HD Detection Reagent-RED kit (#322360, ACDbio, Newark, CA, USA) was used for RNAscope *in situ* hybridization following the manufacturer’s instructions. FFPE tissue sections were baked at 60°C for 1 h and then deparaffinized. The sections were treated with Hydrogen Peroxide (#322381, ACDbio) for 10 min at r.t. After washing twice with deionized water (DW) for 30 s at r.t., target retrieval was performed by heating the sections at 94°C for 15 min in Target Retrieval Reagent (#322000, ACDbio). After another 30 s wash with DW at r.t., the sections were dehydrated in 100% ethanol for 2 min at r.t. and dried at 60°C for 5 min. Next, the sections were treated with Protease Plus (#322381, ACDbio) for 30 min at 40°C in a moisture chamber within a hybridization incubator and washed twice with DW for 1 min at r.t. The RNAscope probes, including positive control (*Ppib*, #313911), negative control (*Dapb*, #310043), *Vglut1* (*Slc17a7*, #416631), *Vglut2* (*Slc17a6*, #319171), and *Vgat* (*Slc32a1*, #319171) (ACDbio), were pre-warmed at 40°C for 10 min and then hybridized to the tissue sections for 2 h at 40°C in the moisture chamber. After hybridization, the sections were washed with Wash Buffer (#310091, ACDbio) for 2 min at r.t. and then proceeded to signal amplification. For this step, the sections were sequentially incubated with AMP1 for 30 min, AMP2 for 15 min, AMP3 for 30 min, AMP4 for 15 min, AMP5 (containing preamplifiers and amplifiers) for 30 min, and AMP6 for 15 min at 40°C in a moisture chamber, following the manufacturer’s instructions. Color development was performed using a mixture of RED-A and RED-B solutions at a 60:1 ratio for 10 min at r.t. in the dark. The development was stopped by washing with tap water for 2 min. For counterstaining, the sections were stained with hematoxylin. To enhance contrast between the reddish color developed by the kit and the reddish color of hematoxylin staining, the color of hematoxylin was converted to blue by soaking the sections in 0.02% ammonia for 10 s at r.t. Finally, the tissue sections were dehydrated at 60°C for 15 min, soaked twice in xylene for 3 s each, and mounted with Mount-Quick.

### Quantitative analysis of RNAscope staining with deep learning

RNAscope staining bright field images were captured using a Nano Zoomer 2.0RS digital slide scanner (Hamamatsu Photonics, Hamamatsu, Japan). The virtual slide images were read and cropped with QuPath, an open-source image analysis software. Four brain regions were used as indicators for cropping thalamus images: the hippocampus (dorsal border), the third ventricle (medial border), the reticular nucleus (RT) (lateral border), and the zona incerta (ventral border). The cropped images were further divided into 500 × 500 pixel (112.7 × 112.7 μm) parcels for annotation and analysis. The number of parcels covered the entire thalamus: 15–18 parcels for the mediolateral axis and 17 parcels for the dorsoventral axis.

To develop a deep learning model for classifying RNAscope-positive and negative cells, Microsoft VoTT (Visual Object Tagging Tool, Microsoft, Redmond, WA, USA) was used for annotation. Sections counterstained with hematoxylin allowed identification of RNAscope-positive cells by red punctate signals around hematoxylin-labeled nuclei. A total of 366 images were annotated, identifying 2,929 RNAscope-positive cells and 14,115 negative cells, resulting in 17,044 cells. The annotated dataset was exported to Roboflow (ver1.1.44, Roboflow Inc., Iowa, USA) to create training, validation, and test splits: 70% for training, 20% for validation, and 10% for testing.

The computer-assisted object detection and classification algorithm, You Only Look Once version 5 (YOLOv5) (Jocher et al., 2021; Redmon et al., 2016) was used for precise classification of stained cells. YOLOv5x served as the trained model. The optimal model was selected with a learning rate between 0.01 and 0.1. Model fitness was evaluated using mean average precision (mAP), calculated by averaging the precision (AP) over two classes and/or various intersection over union (IoU) thresholds. IoU thresholds were determined by dividing the area of overlap by the area of union. Two mAP values, using IoU thresholds of 0.5 and 0.5–0.9, were employed and weighted at 0.1 and 0.9, respectively.

### Statistical analysis

Statistical analyses were performed using JMP Pro version 17 (JMP, Cary, NC, USA).

## Results

### Distribution of PrP^Sc^ in brains of prion-infected mice

**Figure 1:**
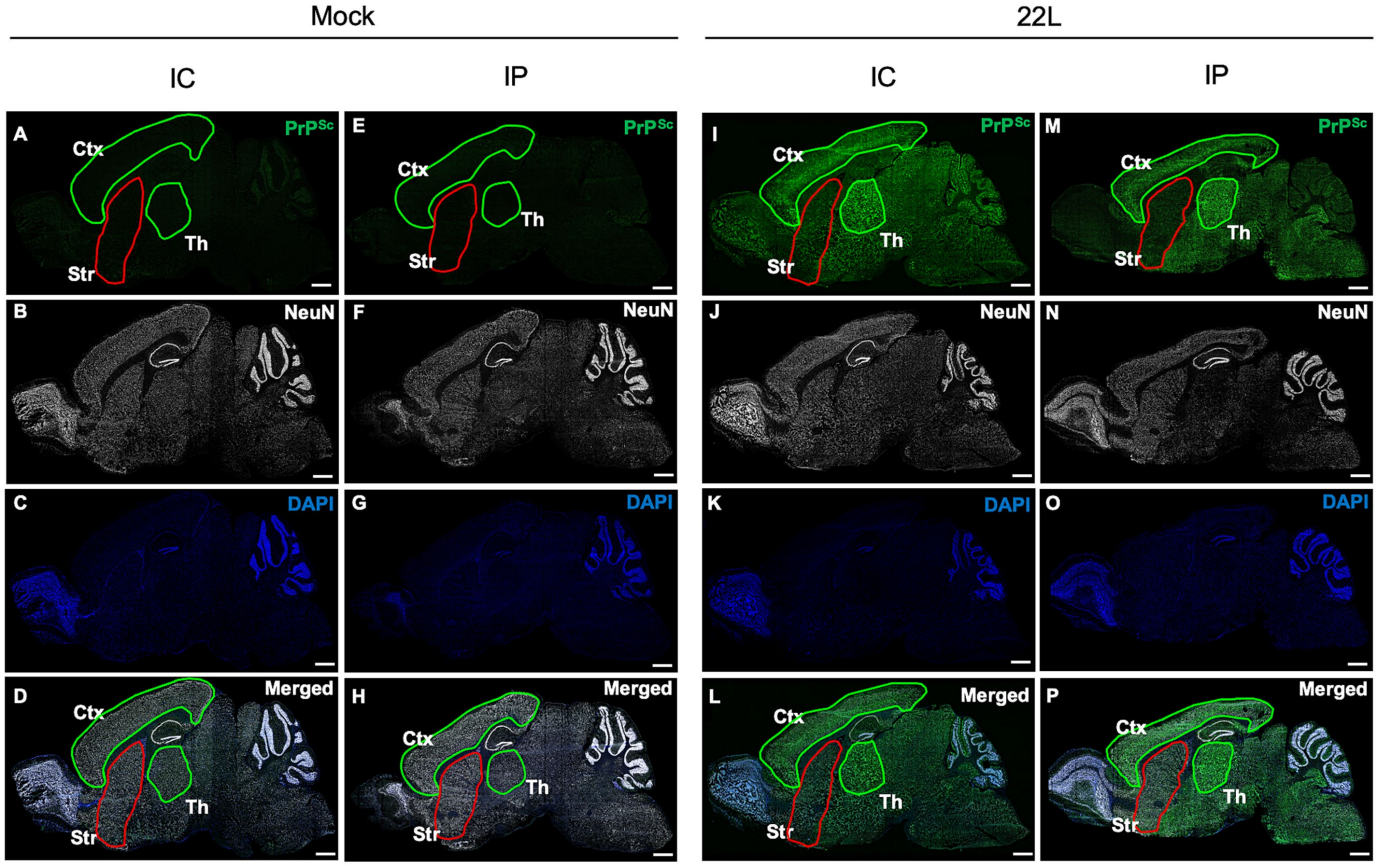
Distribution of PrP^Sc^ in the brains of prion-infected mice. Mice were inoculated intracerebrally (i.c.) or intraperitoneally (i.p.) with 20 μL of 2.5% or 200 μL of 1.0% brain homogenates, respectively, from either mock-infected **(A–H)** or prion 22L strain-infected mice **(I–P)**. Mice were sacrificed at 120 dpi (i.c.) or 186 dpi (i.p.), and their brains were subjected to cryosection. Sagittal sections were cut using a cryostat and stained with anti-PrP mAb 132 to detect PrP^Sc^ (green, **A,E,I,M**) and anti-NeuN mAb for neuronal cells (white, **B,F,J,N**). Nuclei were counterstained with DAPI (blue, **C,G,K,O**). Merged images are shown **(D,H,L,P)**. Images were acquired using a 20× objective lens and are tile scans of sagittal sections generated with ZEN2009 software [black edition]. The cortex (Ctx) and thalamus (Th) regions are outlined in green, while the striatum (Str) is outlined in red. Scale bars: 1 mm. Sagittal brain sections around Plates 111–113 (Paxinos and Franklin, 2013) were used.

To investigate PrP^Sc^ distribution in prion 22L strain-infected mice brains, we analyzed PrP^Sc^ deposition using sagittal cryosections from mice inoculated i.c. or i.p. with prions, at 120 and 186 dpi, respectively, around the clinical onset (Figure 1). PrP^Sc^ signals were absent in age-matched mock-infected mice (Figures 1A,E), while intense PrP^Sc^ signals were observed in the cerebral cortex and thalamus of i.c. inoculated mice (Figure 1I). Conversely, PrP^Sc^ signals in the striatum were notably weaker than in the cerebral cortex and thalamus (Figure 1I). The PrP^Sc^ distribution in i.p. inoculated mice was similar to that in i.c. inoculated mice; intense signals were seen in the cerebral cortex and thalamus, but weaker in the striatum (Figure 1M). This weak PrP^Sc^ staining in the striatum, regardless of inoculation route, suggests inefficient 22 L prion propagation in the striatum. A similar weaker PrP^Sc^ signal in the striatum compared to the cerebral cortex and thalamus was also observed in mice i.c. inoculated with Chandler and Obihiro strains (Supplementary Figure 1).

**Figure 2:**
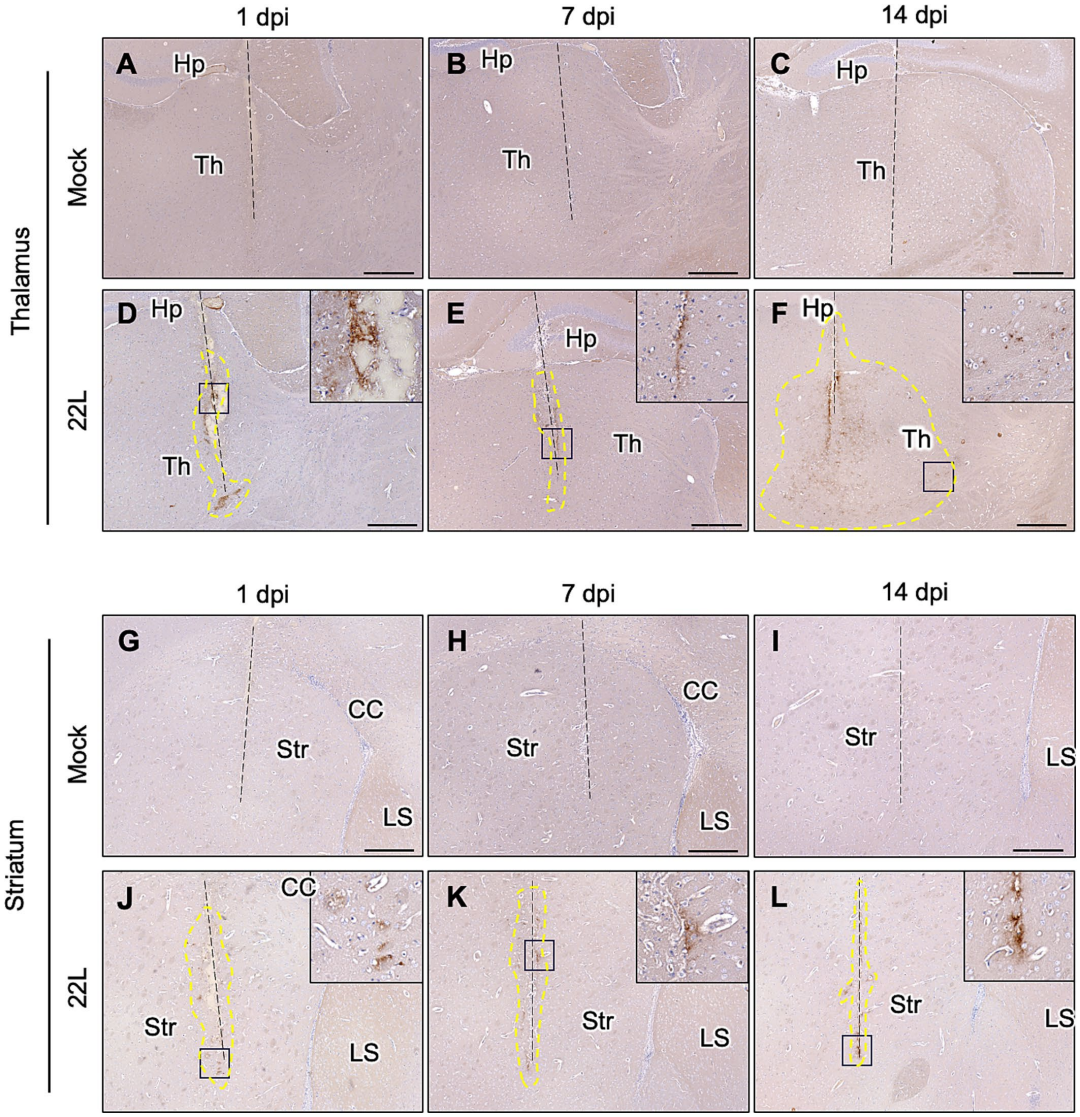
PrP^Sc^ detection after stereotaxic injection of prions. Brain homogenates from mock- or 22L prion-infected mice (0.1%, 0.5 μL) were injected into the thalamus **(A–F)** or striatum **(G–L)** using a stereotaxic apparatus. Brains were collected at 1 **(A,D,G,J)**, 7 **(B,E,H,K)**, and 14 **(C,F,I,L)** days post-injection and fixed with 10% neutral buffered formalin. Coronal sections corresponding to Plates 45–46 (thalamus) and 22–23 (striatum) (Paxinos and Franklin, 2013) were cut for immunohistochemical detection of PrP^Sc^ using anti-PrP mAb 132. FFPE blocks containing either the thalamus or striatum were serially cut into 4 μm sections at the coronal plane using a microtome. Sections in which needle tracks were observed were used for PrP^Sc^ staining with IHC. Dashed lines indicate needle tracks. Higher magnifications of boxed regions in the 22L strain-injected thalamus and striatum are shown in the upper-right corners of each lower magnification image. The region surrounded by dotted lines in the 22 L strain-injected thalamus **(F)** indicates the spread of PrP^Sc^-positive regions from the needle track. Th, thalamus; Hp, hippocampus; Str, striatum; CC, corpus callosum; LS, lateral septum. Scale bars: 300 μm. Dotted lines indicate regions with positive signals determined using ImageJ (ver 1.8.0).

The weak PrP^Sc^ deposition in the striatum suggests that prions propagate more efficiently in the cerebral cortex and thalamus. To further analyze this, we stereotaxically injected 22L prions into either the thalamus or striatum and analyzed PrP^Sc^ deposition by IHC (Figure 2). No visible PrP^Sc^-positive stains were observed in the thalamus and striatum after inoculating age-matched mock-infected brain homogenates (Figures 2A–C,G–I). In the thalamus, positive signals were observed along the needle track at 1 dpi, likely from the PrP^Sc^ in the inoculum (Figure 2D). By 14 dpi, diffuse PrP^Sc^ stains were observed both along and away from the needle track (Figure 2F). Conversely, in the striatum, PrP^Sc^ stains were observed mainly along the needle tracks up to 14 dpi (Figures 2J–L). These results suggest that 22L prions propagate more in the thalamus than in the striatum. We did not analyze prion propagation in the cerebral cortex due to its insufficient thickness for stereotaxic injection.

### Distribution of glutamatergic excitatory and GABAergic inhibitory neurons in mouse brain

**Figure 3:**
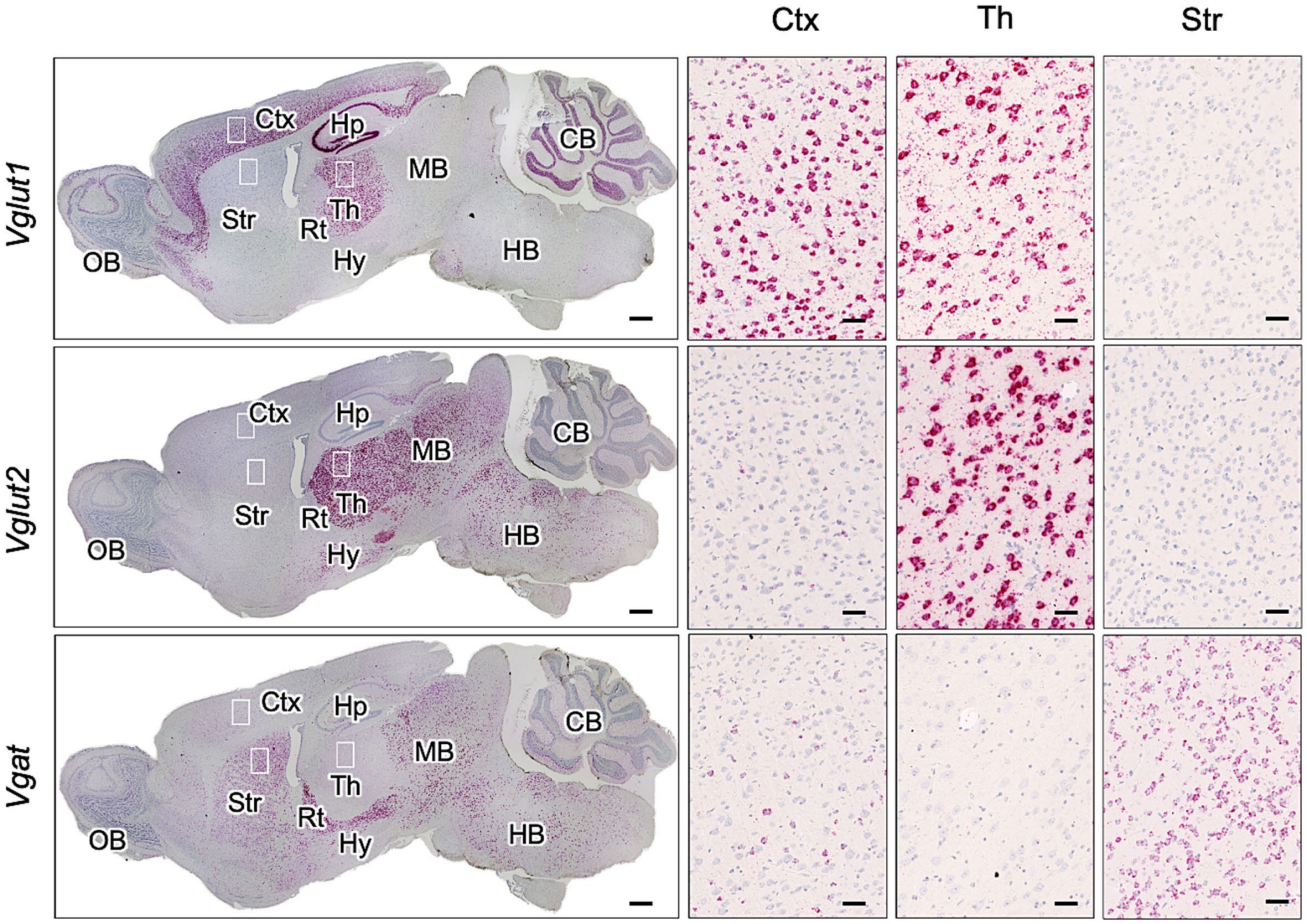
Expression of *Vglut1*, *Vglut2*, and *Vgat* mRNA in mouse brain. RNAscope *in situ* hybridization was used to detect the gene expression of *Vglut1* and *Vglut2*, markers for glutamatergic neurons, and *Vgat*, a marker for GABAergic neurons, in sagittal brain sections from an uninfected adult ICR mouse. The leftmost column displays a whole sagittal section, while the three images on the right show magnified views of the boxed regions in the cortex (Ctx), thalamus (Th), and striatum (Str). Positive signals for the target RNA appear reddish, developed with RNAscope^®^ 2.5 HD Detection Reagent (RED), while counterstaining with hematoxylin produces light blue staining. Images were captured with a NanoZoomer 2.0RS (Hamamatsu Photonic K.K., Hamamatsu, Japan) using a 40× objective and stitched with NanoZoomer 2.0RS software (Hamamatsu Photonic K.K., Hamamatsu, Japan). Scale bars are 1 mm in the sagittal plane images and 0.1 μm in the magnified images. OB, olfactory bulb; Hp, hippocampus; Rt, reticular nucleus; Hy, hypothalamus; MB, midbrain; HB, hindbrain; CB, cerebellum.

Reports indicate that the cerebral cortex has a high proportion of Vglut1-positive excitatory neurons, while Vglut1 or Vglut2-positive neurons are present in the thalamus, and Vgat-positive inhibitory neurons predominate in the striatum (Fremeau et al., 2004b; Rudy et al., 2011). Given the inverse relationship between GABAergic neuron distribution and PrP^Sc^ deposition in the striatum (Figure 1), we first confirmed the gene expression of *Vgat* (a marker for GABAergic inhibitory neurons) and *Vglut1* and *Vglut2* (markers for glutamatergic excitatory neurons) in mouse brains using RNAscope *in situ* hybridization (Figure 3). *Vglut1*-positive cells were mainly observed in the cerebral cortex, pyramidal cell layers, dentate gyrus of the hippocampus, thalamus, and granular cell layer of the cerebellum. *Vglut2*-positive cells were primarily found in the thalamus and midbrain. On the contrary, *Vgat*-positive cells were predominantly located in the striatum and reticular nucleus of the thalamus, with additional presence in the midbrain and sparse distribution in the cerebral cortex (Figure 3).

### Prion propagation in primary neuronal cultures from cortex, thalamus, and striatum

The predominance of GABAergic neurons (Figure 3) and the inefficient 22 L prion propagation in the striatum (Figures 1, 2) suggest a neuronal cell-type-dependent propagation efficiency of the 22 L strain. To investigate this, we prepared primary neuronal cultures from cortices (CxN), thalami (ThN), and striata (StN) to analyze prion propagation *in vitro* based on neuronal cell type.

**Figure 4:**
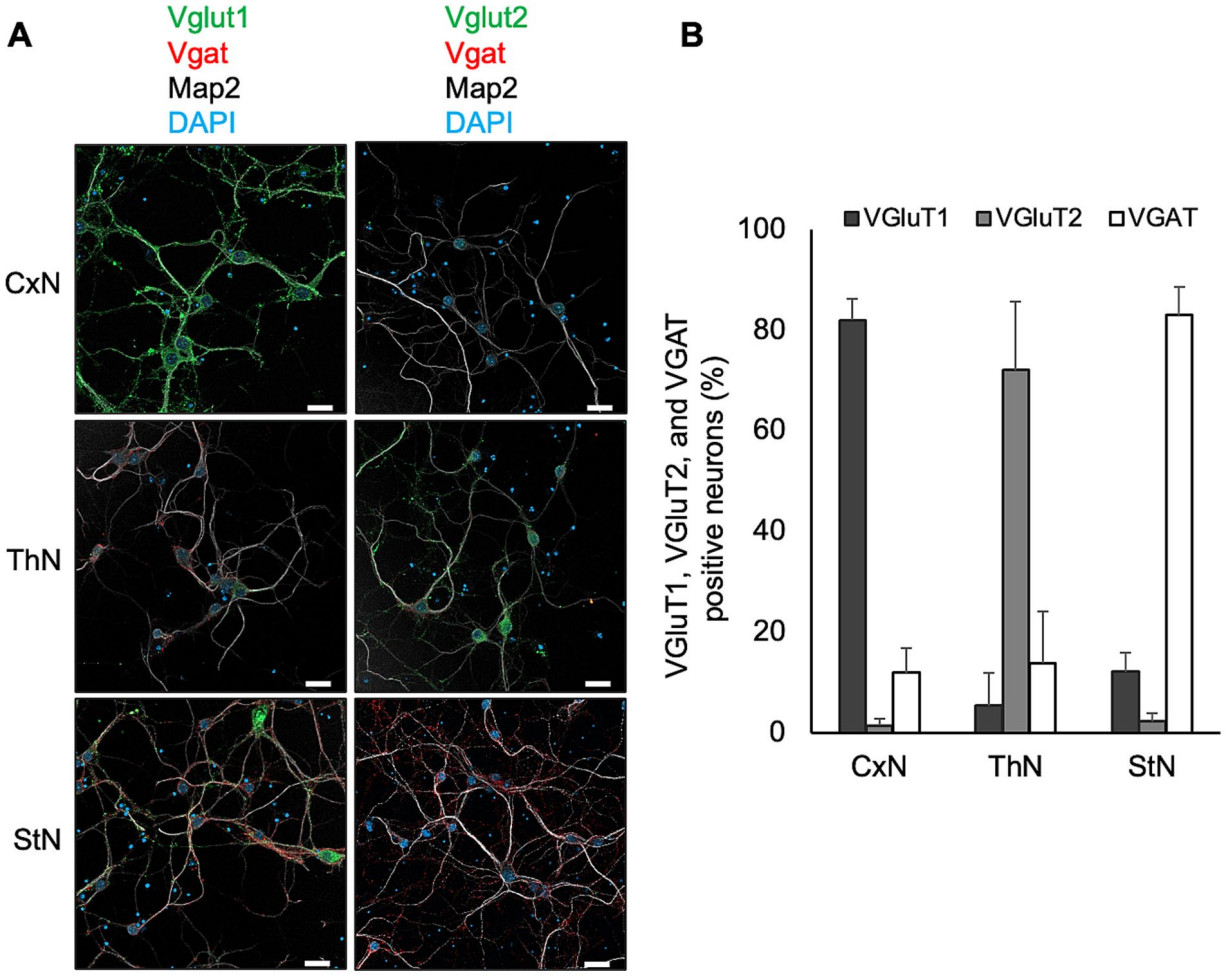
Expression of Vglut1, Vglut2, and Vgat in primary neuronal cultures. **(A)** Expression of markers for glutamatergic excitatory and GABAergic inhibitory synapses in primary neuronal cultures from cortices (CxN, top), thalami (ThN, middle), and striata (StN, bottom). Neuronal cell bodies and dendrites were visualized with anti-MAP2 polyclonal antibody (pAb) (gray). Glutamatergic excitatory synaptic markers were stained with anti-Vglut1 (green, left column) or Vglut2 (green, right column), while GABAergic inhibitory synaptic markers were detected with anti-Vgat (red). Scale bars: 20 μm. **(B)** Proportion of Vglut1-, Vglut2-, and Vgat-positive cells in CxN, ThN, and StN. The graph shows the mean ± SD of six independent experiments. A total of 277, 225, and 398 cells from CxN, ThN, and StN, respectively, were analyzed. Total cell numbers included cells that appeared negative for Vglut1, Vglut2, and Vgat: 14 in CxN, 20 in ThN, and 11 in StN.

First, we examined the expression of Vglut1 and Vglut2, synaptic vesicle membrane transporters specific to glutamatergic excitatory neurons, and Vgat, a synaptic GABA transporter of GABAergic and glycinergic neurons, in each primary neuronal culture using immunofluorescence assay to assess if the proportion of Vglut1-, Vglut2-, and Vgat-positive neurons in the cultures reflects their proportion in the brain regions from which the tissues were collected (Figure 4). Vglut1-positive neurons were predominantly observed in CxN, with a few Vglut2- and Vgat-positive neurons also present. In ThN, Vglut2-positive neurons were mainly observed, with some Vgat-positive neurons. Conversely, Vgat-positive neurons were the most abundant in StN (Figures 4A,B). Since the proportion of Vglut1-, Vglut2-, and Vgat-positive neurons in each neuronal culture from mouse embryos appears roughly consistent with their proportions in adult mouse brains (as shown by RNAscope analysis in Figure 3), we used CtN, ThN, and StN to analyze whether prion propagation differs depending on neuronal cell type.

**Figure 5:**
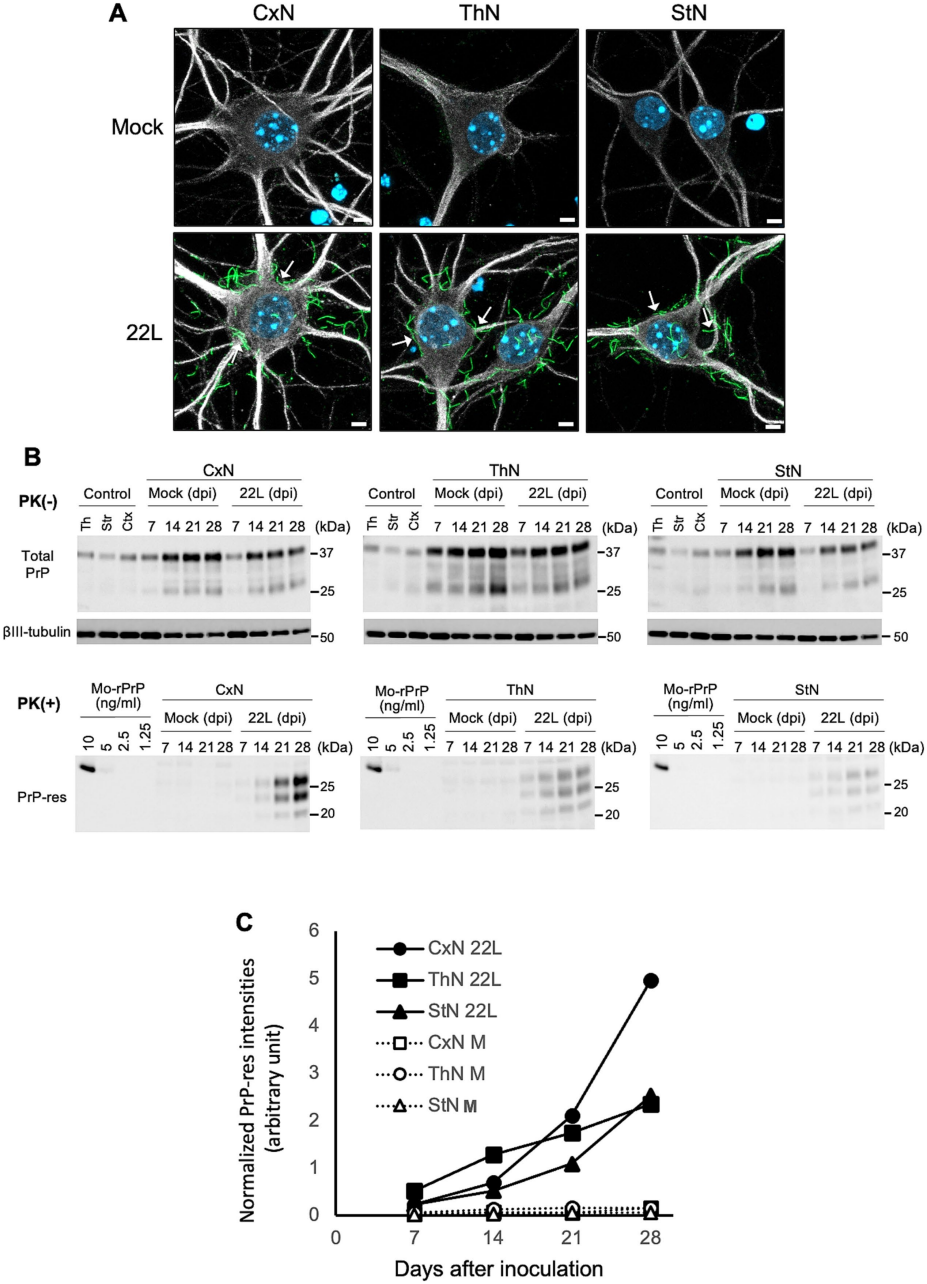
Detection of PrP^Sc^ in primary neuronal cultures infected with prions **(A)** PrP^Sc^ detection in 22L prion-infected neuronal cultures using PrP^Sc^-specific immunostaining with mAb 8D5 (green) at 14 dpi. Upper and lower panels show mock-infected and 22L prion-infected primary neuronal cultures from cortices (CxN), thalami (ThN), and striata (StN), respectively. Arrows indicate string-like PrP^Sc^ strains. Neuronal dendrites were visualized with anti-MAP2 pAb (#ab18207, Abcam) staining (gray), and nuclei were counterstained with DAPI (blue). Scale bars: 5 μm. **(B)** Representative immunoblot images of total PrP, βIII-tubulin, and PrP-res in mock- and prion 22L strain-infected primary neuronal cultures at 7, 14, 21, and 28 dpi. Cell lysates without PK treatment (PK (−)) were used to detect total PrP (mAb 31C6) and βIII-tubulin, while PK-treated cell lysates (PK (+)) were used to detect PrP-res (mAb 31C6). Recombinant PrP (rPrP) was used for quantification of PrP-res and standardization among different blots. Controls represent lysates of each neuronal culture at 21 days *in vitro*. Molecular markers on the right are in kDa. **(C)** Quantification of PrP-res in **(B)**. The amount of PrP-res in different blots was standardized using rPrP intensities, and PrP-res levels were normalized with corresponding βIII-tubulin signal intensities. Results from two independent experiments are shown.

We previously reported that anti-PrP mAb 8D5 detects PrP^Sc^ as string-like stains in prion-infected primary neurons under non-denatured conditions (Tanaka et al., 2016; Tanaka et al., 2020). Figure 5A shows immunofluorescence detection of PrP^Sc^ using mAb 8D5 in neurons exposed to microsomal fractions from 22L-infected mouse brains. String-like stains were observed in all three neuronal cultures infected with 22L prions, while no PrP^Sc^ stains were seen in mock-infected cultures (Figure 5A). Many of the string-like PrP^Sc^ stains appeared to be outside of primary neurons. Most of PrP^Sc^ in prion-infected primary neurons are located on the cell surface (Tanaka et al., 2020), whereas MAP2 is an intracellular protein interacting microtubules of the cellular cytoskeleton. Thus, the string-like PrP^Sc^ stains appeared apart from MAP2 positive regions is due the difference in the cellular localization of PrP^Sc^ and MAP2. Immunoblotting revealed time-dependent increases in PrP-res from 7 to 28 dpi in CxN, ThN, and StN exposed to 22 L prions, indicating successful prion propagation (Figure 5B). Additionally, PrP-res levels in CxN appeared higher than in ThN and StN (Figure 5C). Although neurons with string-like PrP^Sc^ stains were present in all three cultures, the frequency of such neurons appeared to vary. Therefore, we classified PrP^Sc^-positive neurons based on the number of string-like PrP^Sc^ stains (Tanaka et al., 2020) to compare prion propagation efficiency among neuronal cultures.

**Figure 6:**
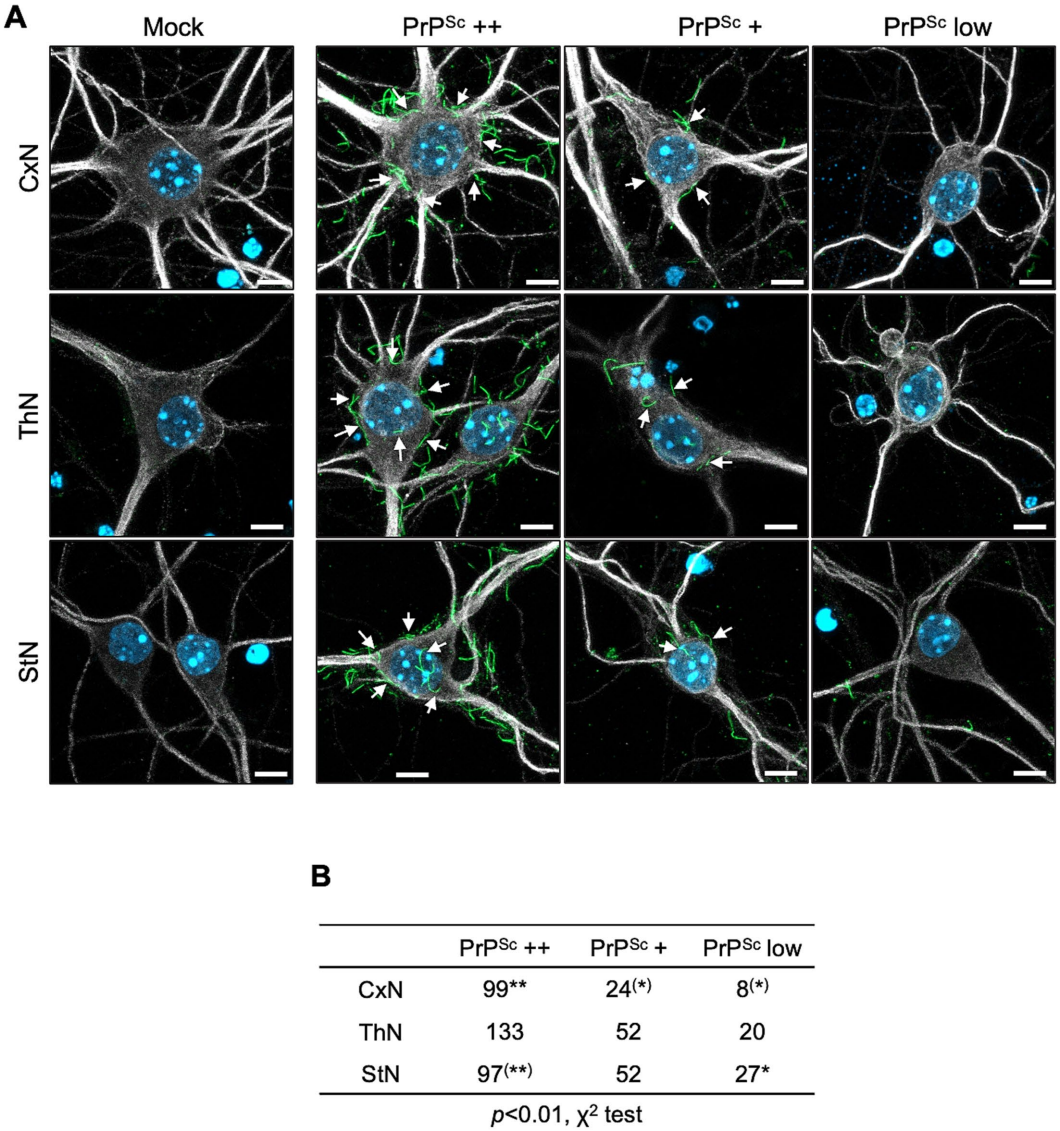
Classification of prion-infected neurons by degree of string-like PrP^Sc^ signals. **(A)** Immunofluorescence detection of PrP^Sc^: Primary cortical (CxN), thalamic (ThN), and striatal (StN) neuronal cultures were infected with the prion 22L strain. At 14 dpi, cultures were stained for PrP^Sc^ using anti-PrP mAb 8D5 (green) and for neuronal bodies and dendrites using anti-MAP2 pAb (gray). Nuclei were counterstained with DAPI (blue). Neurons with five or more, fewer than five, and no apparent string-like PrP^Sc^ signals around the soma were classified as PrP^Sc^ ++, PrP^Sc^ +, and PrP^Sc^ low neurons, respectively. Mock indicates mock-infected cultures. Arrows highlight string-like PrP^Sc^ stains. Scale bars: 5 μm. **(B)** Classification of PrP^Sc^-positive neurons: A total of 131 neurons from CxN (9, 54, 68: cell numbers per experiment), 205 neurons from ThN (39, 87, 79), and 176 neurons from StN (19, 81, 76) were classified into PrP^Sc^ ++, PrP^Sc^ +, and PrP^Sc^ low categories across three experiments. Statistical analysis was performed using the χ^2^ test, followed by residual analysis. ** and ^(^**^)^ indicate items with higher and lower than expected counts, respectively (*p* < 0.01), while * and ^(^*^)^ indicate items with higher and lower expected counts, respectively (*p* < 0.05), by residual analysis.

Figure 6A illustrates the classification of PrP^Sc^-positive neurons based on the number of string-like PrP^Sc^ signals on the surface of the soma. Neurons with five or more, fewer than five, and no apparent string-like PrP^Sc^ signals surrounding the soma were categorized as PrP^Sc^ ++, PrP^Sc^ +, and PrP^Sc^ low neurons, respectively (Figure 6A). We conducted three independent experiments, classifying a total of 131, 205, and 176 neurons from CxN, ThN, and StN, respectively, into these categories (Figure 6B). The frequency of PrP^Sc^ ++, PrP^Sc^ +, and PrP^Sc^ low neurons varied significantly among CxN, ThN, and StN (χ^2^ test, *p* < 0.05). The frequency of PrP^Sc^ ++ neurons was significantly higher than the expected count in CxN, while PrP^Sc^ + and PrP^Sc^ low neurons were significantly lower than the expected count. In contrast, StN had significantly lower frequencies of PrP^Sc^ ++ and higher frequencies of PrP^Sc^ low neurons (residual analysis following χ^2^ test). These results suggest that the 22L strain propagates more efficiently in CxN than in StN.

### Prion propagation in glutamatergic and GABAergic primary neurons

The predominance of Vglut1-positive neurons in CxN aligns with the abundance of Vglut1-positive neurons in the cerebral cortex, while the predominance of Vgat-positive neurons in StN corresponds with the abundance of GABAergic neurons in the striatum. These observations suggest that the propagation efficiency of the 22L strain differs between glutamatergic excitatory and GABAergic inhibitory neurons. To explore this possibility, we first distinguished glutamatergic neurons from GABAergic neurons using the STAIN Perfect Immunostaining Kit A (Supplementary Figure 2). The kit identified glutamatergic neurons as L-glutamate-positive but GABA-negative, and GABAergic neurons as both L-glutamate- and GABA-positive. Most neurons in the CNS use L-glutamate as an excitatory neurotransmitter, while GABA is used as an inhibitory neurotransmitter in inhibitory neurons (Amara and Kuhar, 1993). As expected, most neurons in primary cultures were divided into two populations: L-glutamate-positive but GABA-negative neurons (Supplementary Figure 2, upper panel) and L-glutamate- and GABA-positive neurons (Supplementary Figure 2, lower panel). CxN comprised approximately 70% glutamatergic and 23% GABAergic neurons; ThN had about 62% glutamatergic and 29% GABAergic neurons, while StN consisted of roughly 71% GABAergic and 24% glutamatergic neurons (Supplementary Figure 2).

**Figure 7:**
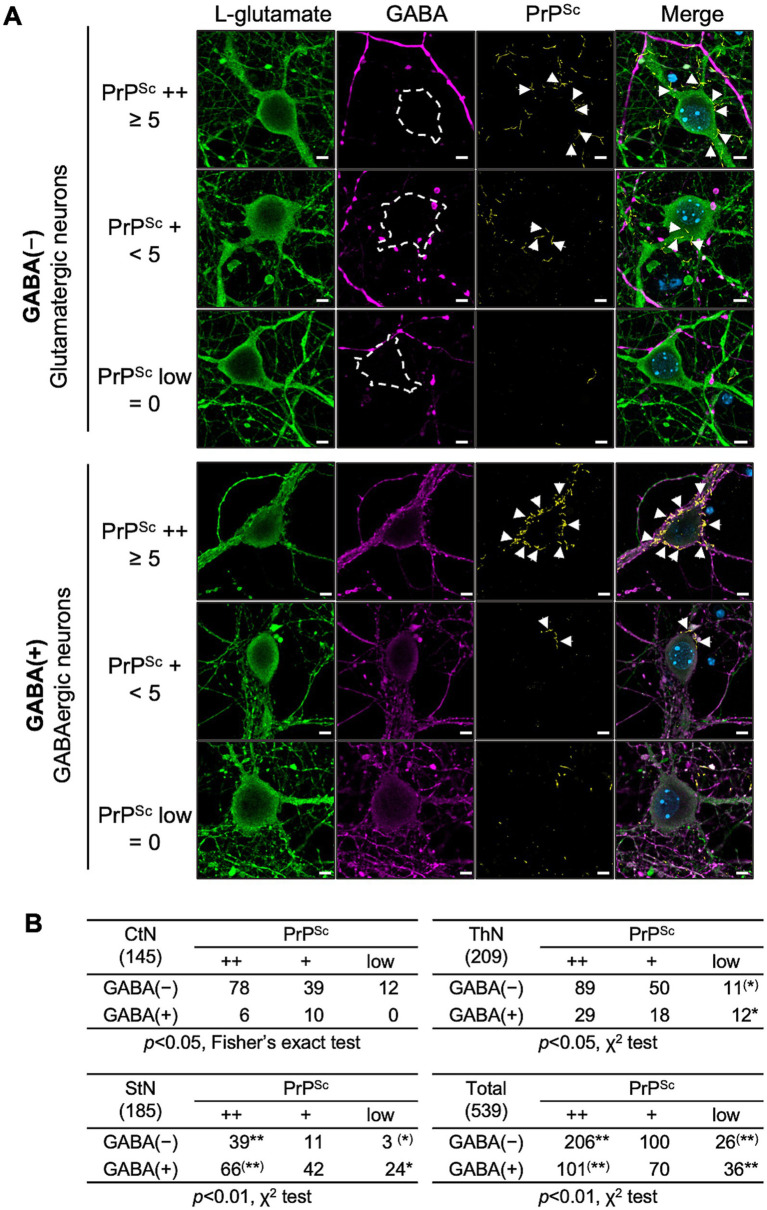
PrP^Sc^ staining with glutamatergic and GABAergic neurons. **(A)** Representative images: At 14 dpi, primary neurons infected with the prion 22L strain were co-stained with anti-L-glutamate Ab (green, leftmost column), anti-GABA Ab (violet, second column from left), and anti-PrP mAb 8D5 (yellow, second column from right). Nuclei were counterstained with DAPI (blue). The rightmost column shows merged images. Representative images of PrP^Sc^ ++, PrP^Sc^ +, and PrP^Sc^ low glutamatergic neurons (upper panel) and GABA-negative GABAergic neurons are shown. Classification of PrP^Sc^ ++, PrP^Sc^ +, and PrP^Sc^ low is described in Figure 6. Arrows indicate string-like PrP^Sc^ stains. Scale bars: 5 μm. **(B)** Comparison of PrP^Sc^-positive neurons: Tables show the numbers of PrP^Sc^ ++, PrP^Sc^ +, and PrP^Sc^ low cells in GABA-negative and GABA-positive neurons in CxN, ThN, and StN. A total of 145 CxN (48, 47, 50: cell numbers per experiment), 209 ThN (41, 107, 61), and 185 StN (57, 77, 51) were analyzed. Statistical analysis was performed using the χ^2^ test, followed by residual analysis. ** and ^(^**^)^ indicate items with higher and lower than expected counts, respectively (*p* < 0.01), while * and ^(^*^)^ indicate items with higher and lower than expected counts, respectively (*p* < 0.05) by residual analysis. Additional 10 sets of images both from PrP^Sc^++ GABA-negative L-glutamate-positive neurons and PrP^Sc^++ GABA-positive L-glutamate-positive neurons were provided as Supplementary Figures 4, 5, respectively, for better understanding of the staining patterns of PrP^Sc^, L-glutamate, and GABA.

We then performed multiple immunofluorescence staining using the STAIN Perfect Immunostaining Kit A to detect L-glutamate and GABA, along with mAb 8D5 for PrP^Sc^. Figure 7A shows representative images for classifying PrP^Sc^ ++, PrP^Sc^ +, and PrP^Sc^ low neurons in either glutamatergic (upper panel) or GABAergic neurons (lower panel). Neurons were categorized based on the string-like PrP^Sc^ stains around the soma, as in Figure 6. A total of 145, 209, and 185 neurons in CxN, ThN, and StN, respectively, were analyzed (Figure 7B). The frequencies of PrP^Sc^ ++, PrP^Sc^ +, and PrP^Sc^ low neurons in GABA-negative and GABA-positive neurons in CxN differed significantly (*p* < 0.05, Fisher’s exact test). Significant differences were also observed in ThN (*p* < 0.05, χ^2^ test), with residual analysis showing a significantly lower frequency of PrP^Sc^ low GABA-negative neurons and a significantly higher frequency of PrP^Sc^ low GABA-positive neurons (*p* < 0.05). Significant differences were also observed in StN (*p* < 0.01, χ^2^ test); the frequency of PrP^Sc^ ++ GABA-negative neurons was significantly higher, while that of PrP^Sc^ ++ GABA-positive neurons was significantly lower than the corresponding expected counts. Given the varying proportions of GABA-negative and GABA-positive neurons among the three neuronal cultures, we combined the results and compared the frequencies of PrP^Sc^ ++, PrP^Sc^ +, and PrP^Sc^ low in GABA-negative and GABA-positive neurons (Figure 7B). The frequencies of PrP^Sc^ ++ neurons were significantly higher than the expected count in GABA-negative neurons and lower than the expected count in GABA-positive neurons (*p* < 0.01). Conversely, the frequencies of PrP^Sc^ low neurons were significantly lower in GABA-negative neurons and higher in GABA-positive neurons (*p* < 0.01). These findings indicate that the 22 L prion strain propagates more efficiently in GABA-negative glutamatergic neurons than in GABA-positive GABAergic neurons in primary neuronal cultures.

### Type of neurons that are lost in the thalamic nuclei

**Figure 8:**
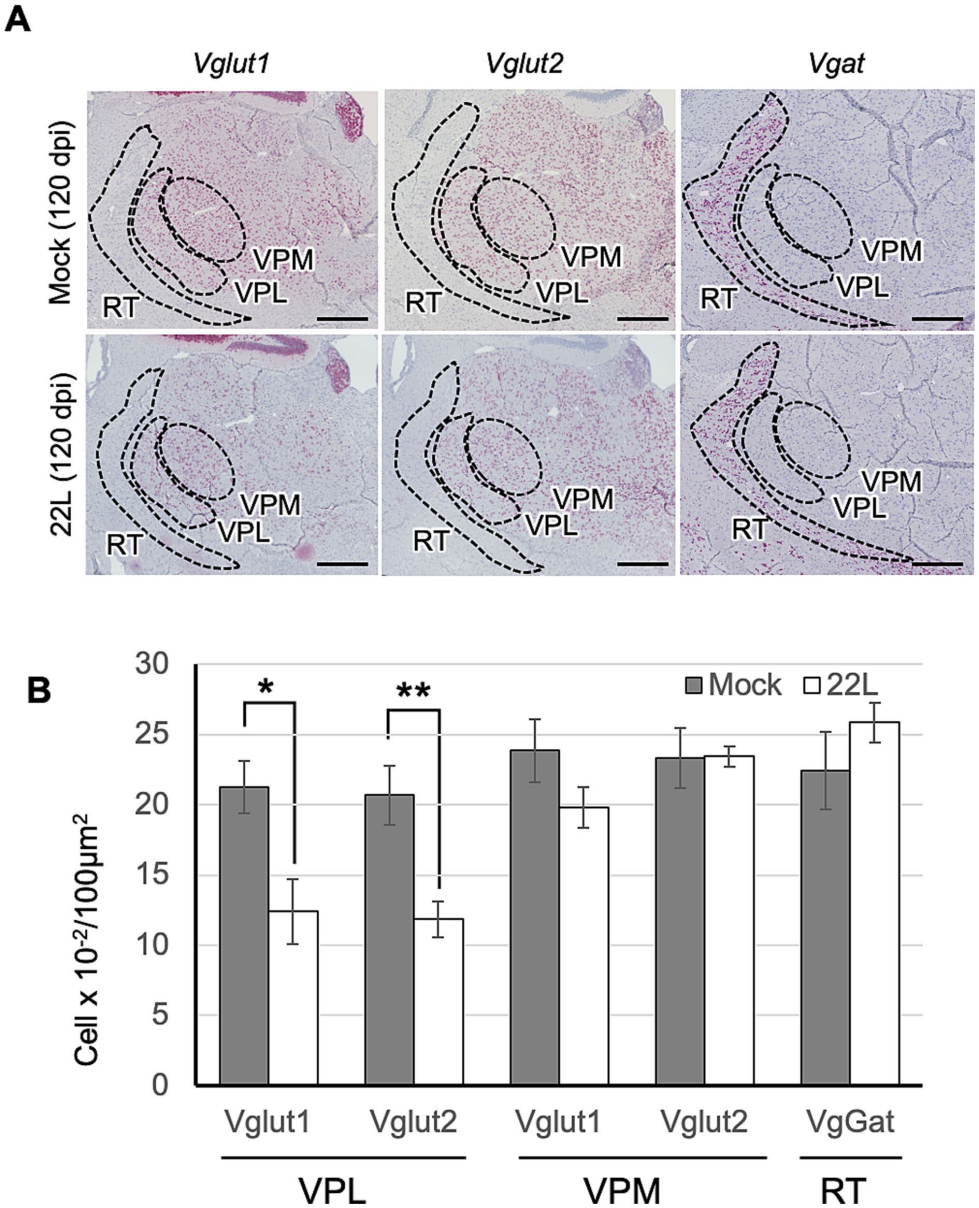
Type of thalamic neurons lost in prion infection. **(A)** RNAscope analysis: RNAscope *in situ* hybridization was used to detect the gene expression of *Vglut1* and *Vglut2*, markers for glutamatergic neurons, and *Vgat*, a marker for GABAergic neurons, in the thalamus of prion 22L strain-infected mice at 120 dpi. Coronal sections around Plate 45–46 (Paxinos and Franklin, 2013) were used. Positive signals for the target RNA were developed with RNAscope^®^ 2.5 HD Detection Reagent—RED, showing a reddish color, while counterstaining with hematoxylin produced light blue staining. Images were captured with a NanoZoomer 2.0RS using a 40× objective and stitched with NanoZoomer 2.0RS software. The ventral posterolateral nucleus (VPL), ventral posteromedial nucleus (VPM), and reticular nucleus (RT) are enclosed with dashed lines. Scale bar: 0.5 mm. **(B)** Quantitative analysis: Neurons positive for *Vglut1*, *Vglut2*, or *Vgat* were counted as described in the materials and methods. Graphs show the number of positive cells per 100 μm^2^ (mean ± SD of 3 brains from either mock- or prion-infected mice). Three sections were analyzed from each mouse. Statistical analysis was performed using Student’s *t*-test, *p < 0.05, **p < 0.01.

Our results indicate that the 22L strain propagates more efficiently in glutamatergic neurons than in GABAergic neurons. We have observed massive neuronal loss in the lateral thalamus of Jcl:ICR mice infected with the 22L, Chandler, and Obihiro strains (unpublished observation), similar to previous reports (Cunningham et al., 2005; Reis et al., 2015). Thus, to identify the types of neurons lost in the lateral thalamus of 22L strain-infected mice, we analyzed *Vglut1*, *Vglut2*, and *Vgat*-expressing cells using RNAscope *in situ* hybridization (Figure 8). *Vglut1* and *Vglut2* expression was observed in both the lateral and medial thalamus (Figure 8A). Compared to age-matched mock-infected mice, the number of *Vglut1-* and *Vglut2*-expressing neurons significantly decreased in the ventral posterolateral nucleus (VPL). In in the ventral posteromedial nucleus (VPM), *Vglut1-*expressing neurons tended to decrease but no apparent decrease in *Vglut2-*expressing neurons was observed in 22L strain-infected mice (Figure 8B). No decrease in *Vgat*-expressing neurons was observed in the reticular nucleus of the thalamus (RT), which is adjacent to VPL and VPM and primarily composed of *Vgat*-expressing interneurons.

## Discussion

The distribution of PrP^Sc^ and neuropathological lesions varies depending on prion strains as well as the combination of prion and mouse strains (Bruce et al., 1994; Bruce, 1993; DeArmond et al., 1993). In this study, we attempted to address the underlying mechanisms using *in vivo* and *ex vivo* experiments. We found that the inefficient propagation of the 22 L strain in GABAergic inhibitory neurons contributes, if not entirely, to the weaker PrP^Sc^ deposition in the striatum compared to the cerebral cortex and thalamus in 22 L strain-infected mice (Figure 1). After stereotaxic inoculation of the 22 L strain into the striatum, which is primarily composed of GABAergic neurons, PrP^Sc^ deposition was observed along the needle tracks. In contrast, diffuse PrP^Sc^ deposits were detected both along and away from the needle track in the thalamus, where excitatory neurons are predominant (Figures 2, 3). The findings indicate that the 22 L strain may propagate more efficiently in the thalamus than in the striatum. PrP^Sc^ deposition along the needle tracks after stereotaxic inoculation into the striatum aligns with previous observations (Chesebro et al., 2015; Rangel et al., 2014). Intracerebrally inoculated prions spread through the brain via interstitial fluid flow (Chesebro et al., 2015), and prions associated with neurons are believed to spread through neuroanatomical pathways (Bartz et al., 2002; Fraser, 1982; Reis et al., 2015). Consequently, PrP^Sc^-positive signals were observed in the striatum, but the PrP^Sc^-positive areas did not spread efficiently after stereotaxic injection, suggesting that neuronal spread of prions through GABAergic inhibitory neurons or transmission across inhibitory synapses is inefficient. Over 50% of neurons in prion-infected StN were still classified as PrP^Sc^ ++, although the frequency of PrP^Sc^ ++ was significantly lower in prion-infected StN than in CxN (Figure 6). This suggests that the 22 L prion might also propagate efficiently in GABAergic neurons, but the weak PrP^Sc^ deposition in the striatum could be due to the inefficient reach of prions after i.c. and i.p. inoculation. The seemingly contradictory differences might be explained by the experimental condition where many neuronal cells are exposed to the microsome fraction during prion inoculation, meaning the efficacy of neuron-to-neuron spread may not be fully reflected in the frequencies of PrP^Sc^ ++ neurons. Further studies are needed to precisely address the efficiency of prion propagation in GABAergic neurons and the transmission efficiency between them.

Since the level of PrP^C^ affects prion propagation (Büeler et al., 1993), we analyzed PrP^C^ levels in three primary neuronal cultures. The level of PrP^C^ in the StN was 47 and 62% of that of PrP^C^ in the CxN and ThN, respectively (Supplementary Figure 3C), suggesting that the lower level of PrP^C^ might be one of the factors in the inefficient prion propagation in StN. However, similar tendency was not observed in the PrP^C^ expression in the striatum of mock-infected mouse (Supplementary Figures 3A,B). PrP^C^ expression and PrP^Sc^ formation is influenced by the cell-to-cell contact (Nakamitsu et al., 2010), and PrP^C^ expression is essential but not a sole determinant for prion permissiveness of neuronal cells (Slota et al., 2023; Uryu et al., 2007). Thus, because of the complexity in brain, it is difficult to assess if the PrP^C^ level of the striatal neurons influences prion propagation efficiency.

Certain subsets of GABAergic inhibitory neurons are reported to be vulnerable in the brains of CJD patients and prion-infected animals (Belichenko et al., 1999; Guentchev et al., 1997; Guentchev et al., 1999). However, these observations are controversial, as some reports indicate that degeneration of GABAergic neurons was observed in the same degenerative sequence of event as non-GABAergic neurons in prion-infected hamsters (Bouzamondo et al., 2000; Bouzamondo-Bernstein et al., 2004). In this study, we analyzed prion propagation and vulnerability in GABA-negative glutamatergic excitatory neurons and GABA-positive GABAergic inhibitory neurons. Using multiple fluorescence staining with L-glutamate, GABA, and PrP^Sc^, we compared prion propagation efficiency in glutamatergic and GABAergic neurons. The frequency of PrP^Sc^ ++ neurons was significantly higher in glutamatergic neurons than in GABAergic neurons, while PrP low neurons were more frequent in GABAergic neurons than in glutamatergic neurons (Figure 6). These results suggest that the 22L strain propagates more efficiently in glutamatergic excitatory neurons than in GABAergic inhibitory neurons. This interpretation aligns with the observation that PrP^Sc^ accumulation is more pronounced in the cerebral cortex and thalamus, regions rich in glutamatergic neurons, compared to the striatum, which is abundant in GABAergic neurons (Figure 3). To our knowledge, this provides the first direct evidence of excitatory neurons being more permissive to prion propagation. In this study, we showed excitatory neuron-prone prion propagation in primary neuronal cultures. Although technical limitations on a finer distinction of PrP^Sc^ stains in neurons and astrocytes in brain sections remain to be resolved, *in vivo* relevance of this phenomenon should be addressed to contribute to elucidating prion neuropathobiology at a higher level.

Massive neuronal losses are observed in the thalamus of mice infected with the 22 L strain and other prion strains (Cunningham et al., 2005; Reis et al., 2015). To identify the specific neuronal types affected, RNAscope *in situ* hybridization was used. It revealed a decrease in both *Vglut1*- and *Vglut2*-positive neurons in the VPL at 120 dpi (Figure 8). However, no decrease in Vgat-positive neurons was observed in the RT thalamic nucleus, which is adjacent to the VPL and primarily composed of GABAergic neurons (Pinault, 2004). Sakaguchi et al. recently reported a selective decrease in Vglut1 staining, but not in Vglut2 and Vgat staining along with the loss of granule cells in the cerebellum and decreased Vglut1 immunoreactivity in the pontine nuclei of BSE-infected guinea pigs (Sakaguchi et al., 2020). Altered Vglut1 staining has also been noted in the retina of scrapie-infected sheep (Smith et al., 2008). Additionally, no loss of parvalbumin-positive GABAergic neurons was observed in the striatum of prion 139A-infected mice (Gunapala et al., 2010) or in the hippocampus of prion ME7-infected mice (Franklin et al., 2008). These findings suggest the vulnerability of excitatory neurons. Conversely, in addition to the loss of parvalbumin-positive GABAergic neurons in CJD patients and experimental animals (Belichenko et al., 1999; Guentchev et al., 1997; Guentchev et al., 1999), recent single-cell transcriptome analysis indicates the vulnerability of inhibitory neurons in the hippocampus to prion infection (Slota et al., 2022). Human cerebral organoids with the familial CJD mutation (PrP E200K) also showed impairment of GABAergic neurons (Foliaki et al., 2021). Thus, while we observed excitatory neuron-prone prion propagation and loss of excitatory neurons in prion-infected mice, we believe that the neuropathogenesis of prion diseases involves a complex balance between excitatory and inhibitory neurons. Since synaptic losses and axonal degeneration occur earlier than neuronal losses in prion diseases (Liberski and Budka, 1999; Reis et al., 2015; Sisková et al., 2009) and acute neurotoxicity caused by the induction of PrP^C^-dependent neurotoxic signals through PrP^C^-PrP^Sc^ interaction appears to be selective to excitatory synapse in the neuronal culture model (Fang et al., 2018; Foliaki et al., 2018), functional analyses of both excitatory and inhibitory neurons are needed to understand the precise neuropathogenesis.

Losses of excitatory neurons and synapses are well-documented in Alzheimer’s disease (Kashani et al., 2008; Mi et al., 2023; Rodriguez-Perdigon et al., 2016), tauopathies (Fu et al., 2017), and Parkinson’s disease (Zhao et al., 2023). Additionally, the loss and dysfunction of inhibitory neurons are implicated in Alzheimer’s disease pathology (Mathys et al., 2023; Palop and Mucke, 2016), though the mechanisms of neuronal dysfunction remain unclear. Understanding the neuropathology of prion diseases, including the mechanisms of neuronal cell-type-prone prion propagation and neuronal vulnerability, will enhance our integrated understanding of neurodegenerative disorders caused by protein misfolding. One advantage of prion disease research is the availability of primary neuronal cultures susceptible to prions. Although apparent neuronal death or synaptic alterations were not observed in prion-infected primary neuronal cultures (Tanaka et al., 2020), enhanced phosphorylation of protein kinase RNA-activated-like endoplasmic reticulum kinase (PERK), an early cellular response of the PERK-eukaryotic initiation factor 2-ATF4 unfolded protein response pathway, was induced in prion-infected primary neurons (Tanaka et al., 2020). This suggests the potential of primary neuronal cultures for analyzing the molecular mechanisms of neurodegeneration caused by prion infection. Further improvement of these cultures as models for prion disease neuropathology will be key to elucidating the molecular mechanisms underlying prion neuropathology.

## Data Availability

The original contributions presented in the study are included in the article/Supplementary material, further inquiries can be directed to the corresponding author.
