# Supplementary material for: Excitatory neuron-prone prion propagation and excitatory neuronal loss in prion-infected mice
Source: Front Mol Neurosci. 2024 Dec 12;17:1498142. doi: 10.3389/fnmol.2024.1498142 (PMC11669680; doi:10.3389/fnmol.2024.1498142)
Supplement: Supplementary file 1 [file Data_Sheet_1.PDF]

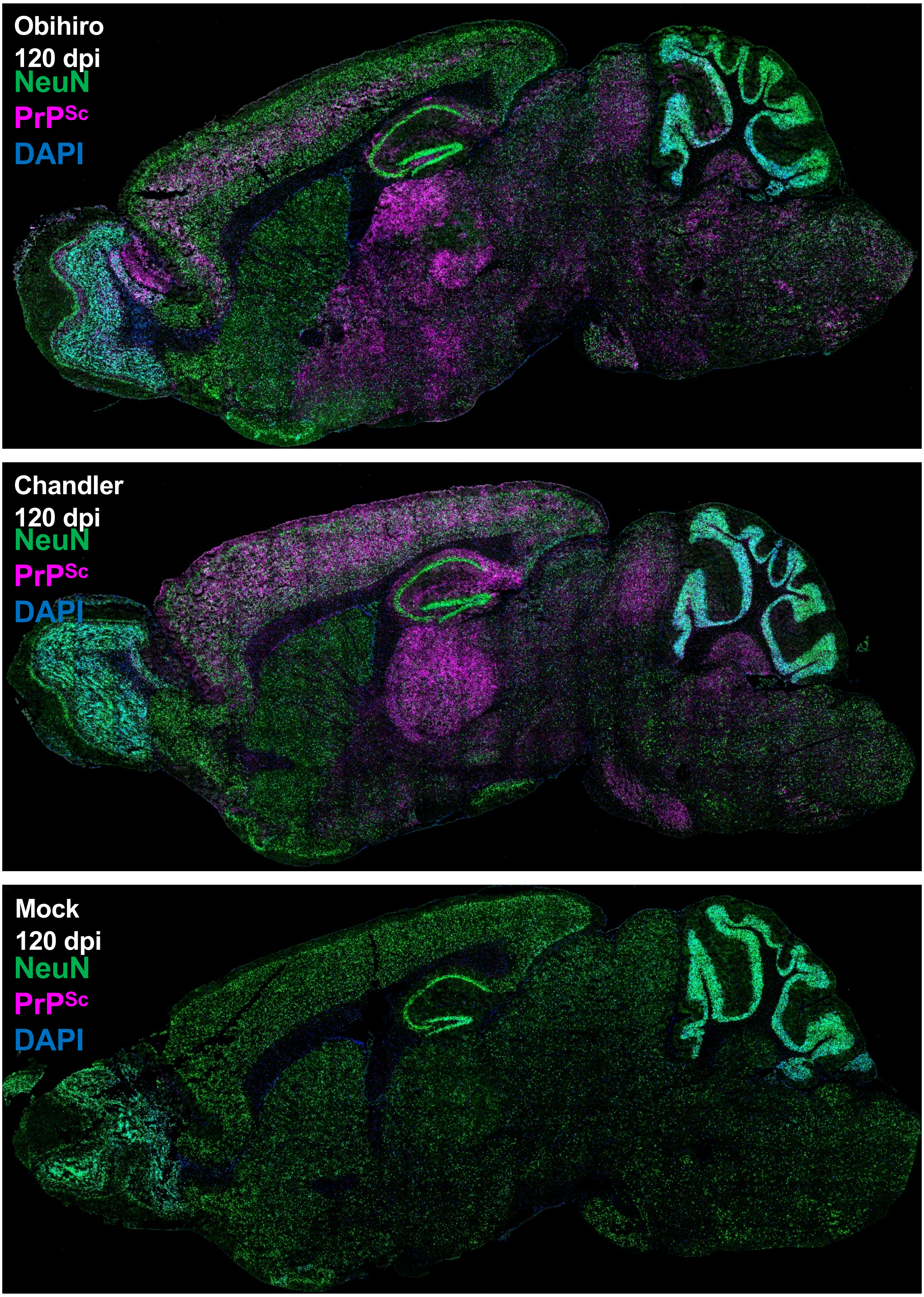

Supplementary Fig. 1. Distribution of PrP^{Sc} in brains of prion Chandler and Obihiro strain-infected mice at 120 dpi.

Mice were intracerebrally (i.c.) inoculated with brain homogenates from mock-, Obihiro-, or Chandler strain-infected mice. At 120 dpi, mice were sacrificed, and their brains were subjected to cryosection. Merged images show anti-NeuN mAb (green), PrP^{Sc} (magenta), and DAPI (blue). Images were acquired using a 20× objective lens and are tile scans of sagittal sections generated with ZEN2009 software (black edition). Sagittal brain sections around Plates 111–113 (Paxinos and Franklin, 2013) were used.

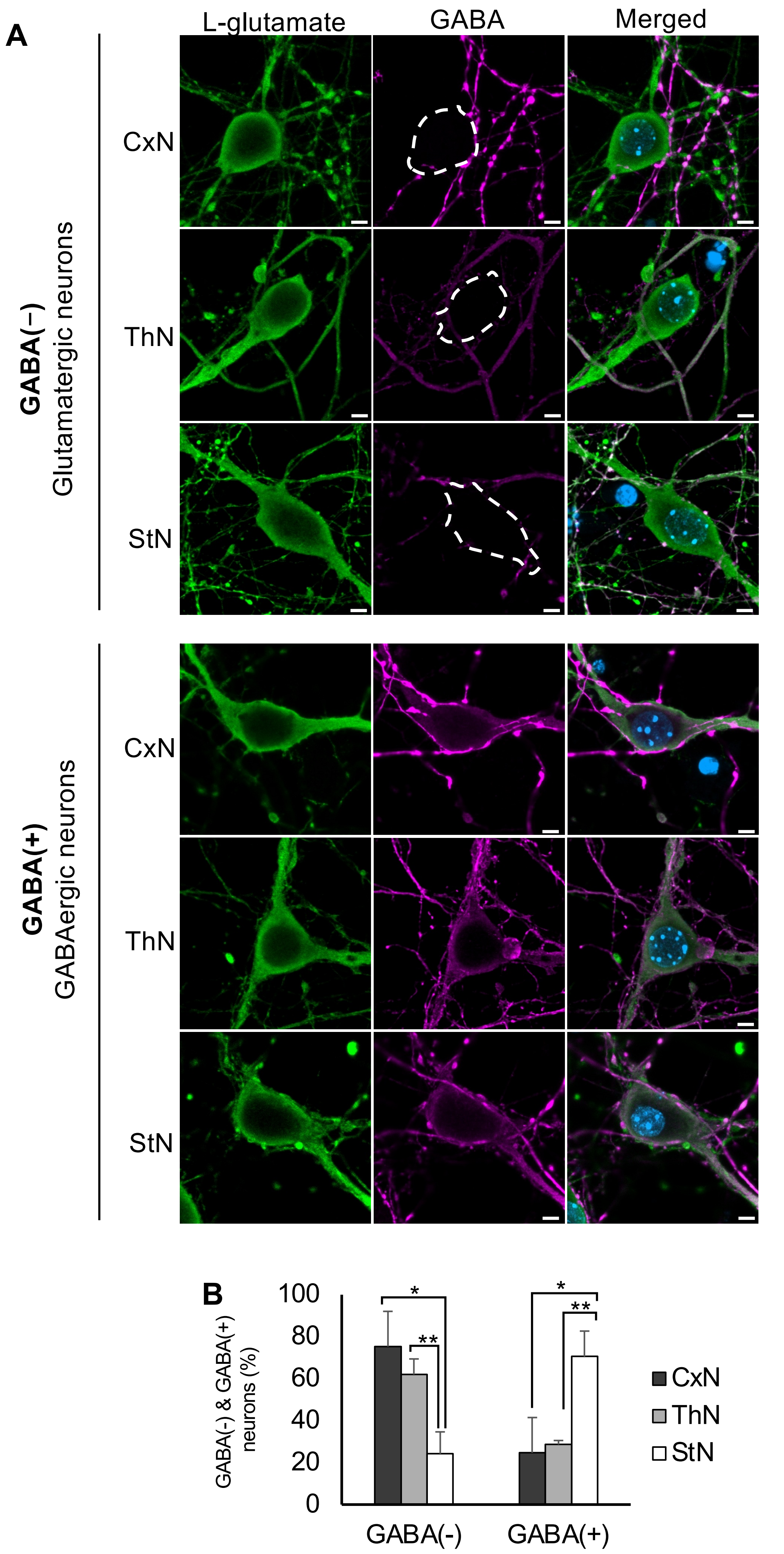

Supplementary Fig. 2. Temuulen et al

Supplementary Fig. 2. Distinction between GABA-negative glutamatergic neurons and GABA-positive GABAergic neurons

(A) Distinction of glutamatergic and GABAergic neurons: Primary cortical (CxN), thalamic (ThN), and striatal (StN) neuronal cultures at 14 dpi were co-stained with anti-L-glutamate (green) and anti-GABA (violet) antibodies using the STAIN Perfect Immunostaining Kit A. Nuclei were counterstained with DAPI (blue) to distinguish GABA-negative glutamatergic neurons from GABA-positive GABAergic neurons. Representative images of GABA-negative glutamatergic neurons (upper panel) and GABA-positive GABAergic neurons (lower panel) from three different neuronal cultures are shown. Cells enclosed by dotted-lines are L-glutamate-positive but GABA-negative. Scale bars: 5 μm. (B) Proportion of GABA-negative glutamatergic and GABA-positive GABAergic neurons: A total of 184, 234, and 190 cells from CxN, ThN, and StN, respectively, were analyzed. Total cell numbers included cells that appeared negative for both glutamate and GABA: 14 in CxN, 22 in ThN, and 10 in StN. Graphs show mean ± SD from three independent experiments. Statistical analysis was performed using Student's *: $p < 0.05$, **: $p < 0.01$.

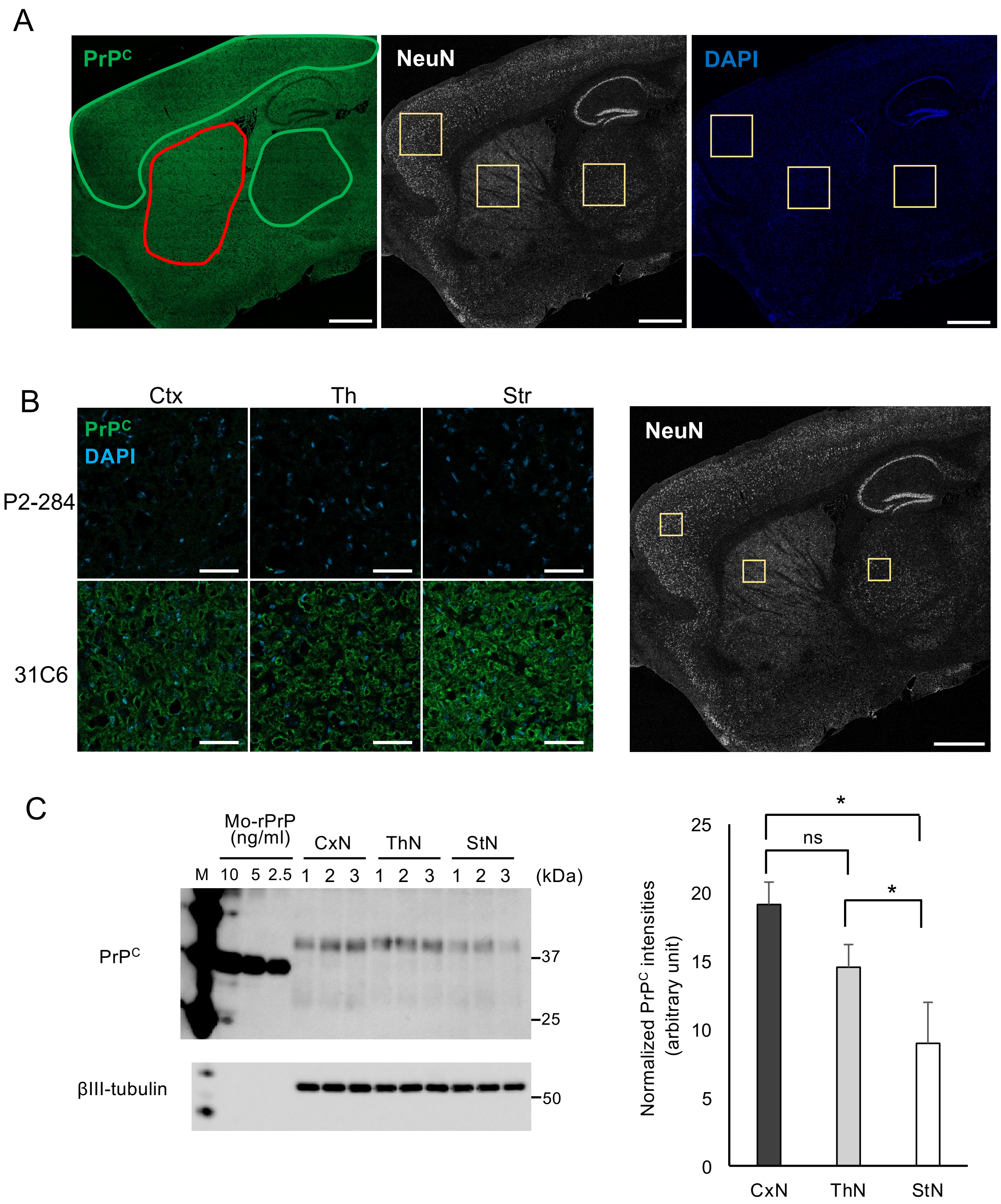

Supplementary Fig. 3. PrPC expression in brain and primary neuronal cultures.

(A) Mock-infected mice (i.c.) were sacrificed at 120 dpi and brains were subjected to cryosection. Sagittal sections were cut using a cryostat and stained with anti-PrP mAb 31C6 to detect PrP^{C} (green) and anti-NeuN mAb for neuronal cells (white). Nuclei were counterstained with DAPI (blue). Images were acquired using a 20× objective lens and are tile scans images were generated with ZEN2009 software [black edition]. The cortex (Ctx) and thalamus (Th) regions are outlined in green, while the striatum (Str) is outlined in red. Scale bars: 1 mm. Sagittal brain sections around Plates 111 to 113 (Paxinos and Franklin, 2013) were used. (B) Sagittal brain sections were stained with anti-PrP mAb 31C6 and negative control mAb (anti-feline parvovirus subgroup mAb P2-284) and regions in the cortex (Ctx), thalamus (Th), and striatum (Str) indicated with boxes in the sagittal section on the right are shown. Scale bars are 50 µm and 1 mm, in the left panel images and in the sagittal plane image, respectively. (C) Immunoblot of total PrP^{C} and βIII-tubulin in primary neuronal cultures from the cortex (CxN), thalamus (ThN), and striatum (StN) at 21 div (in triplicate). The mAb 31C6 was used to detect PrP^{C}. Graph on the right shows quantification of PrP^{C}. Mean and SD are indicated. * p <0.05.

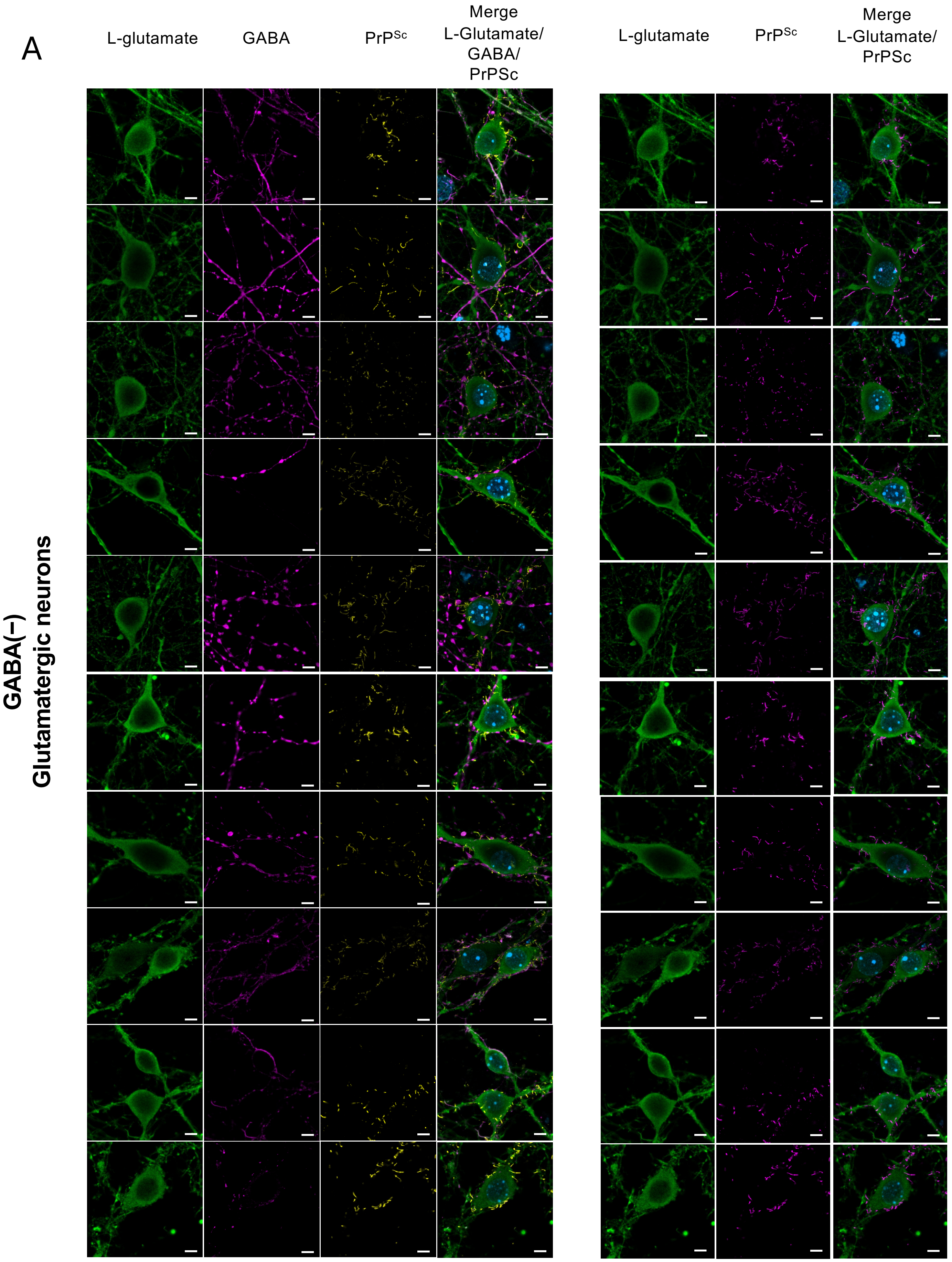

Supplementary Fig. 4. PrP^{Sc} staining with glutamatergic neurons.

(A) Left panel: additional 10 sets of PrP^{Sc}++ GABA-negative L-glutamate-positive glutamatergic neurons are shown. Co-stained with anti-L-glutamate Ab (green, leftmost column), anti-GABA Ab (violet, second column from left), and anti-PrP mAb 8D5 (yellow, second column from right). Nuclei were counterstained with DAPI (blue). Right panel: The same sets in the left panel but violet channel showing GABA-stains were removed and PrP^{Sc} stains were indicated with violet. Scale bars: 5 μm.

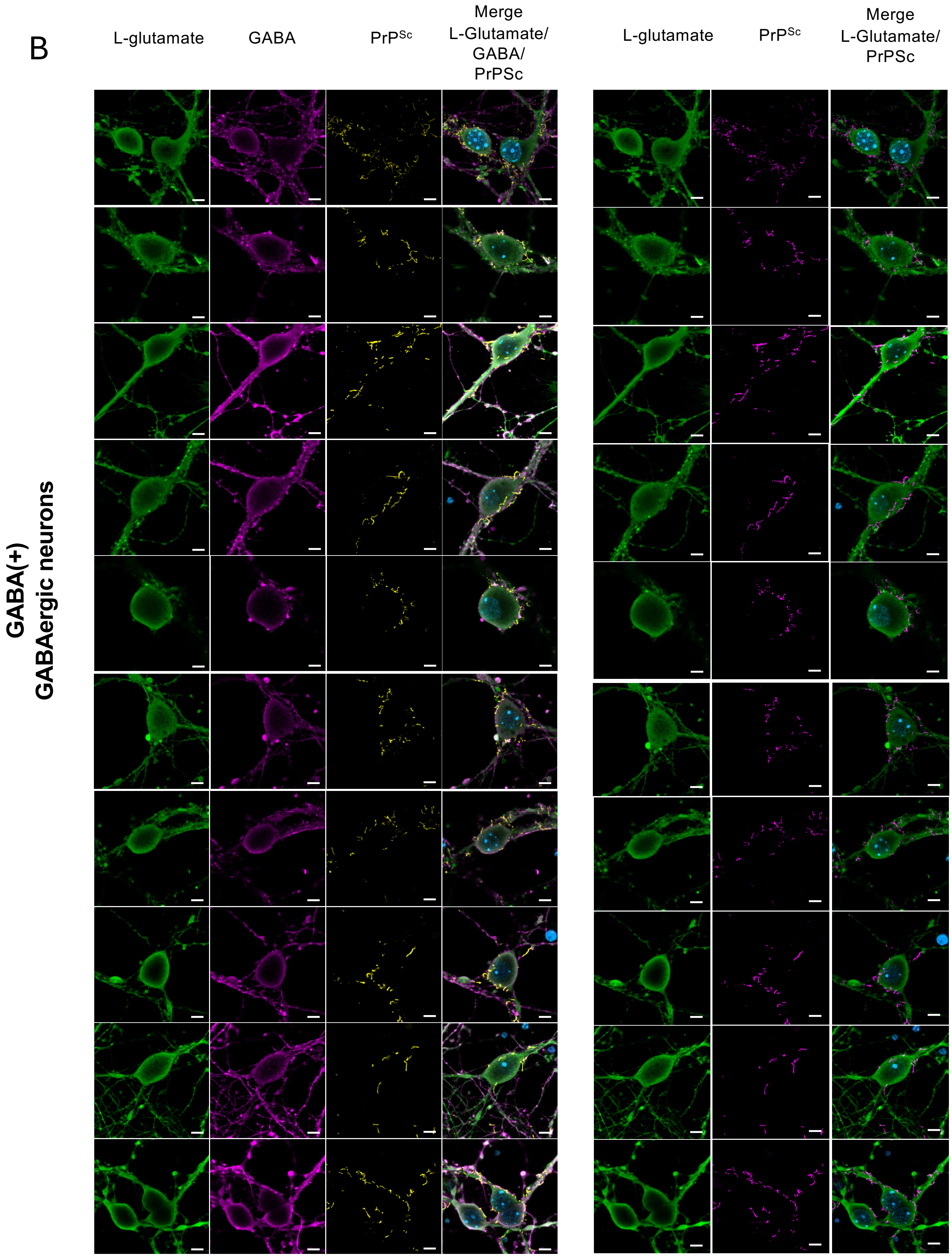

Supplementary Fig. 5. PrP^{Sc} staining with GABAergic neurons.

(A) Left panel: additional 10 sets of PrP^{Sc}++ GABA- and L-glutamate-positive GABAergic neurons are shown. Co-stained with anti-L-glutamate Ab (green, leftmost column), anti-GABA Ab (violet, second column from left), and anti-PrP mAb 8D5 (yellow, second column from right). Nuclei were counterstained with DAPI (blue). Right panel: The same sets in the left panel but violet channel showing GABA-stains were removed and PrP^{Sc} stains were indicated with violet. Scale bars: 5 µm.
